# Reliable quantum certification of photonic state preparations

**DOI:** 10.1038/ncomms9498

**Published:** 2015-11-18

**Authors:** Leandro Aolita, Christian Gogolin, Martin Kliesch, Jens Eisert

**Affiliations:** 1 Dahlem Center for Complex Quantum Systems, Freie Universität Berlin, 14195 Berlin, Germany; 2 Instituto de Física, Universidade Federal do Rio de Janeiro, P. O. Box 68528, Rio de Janeiro 21941-972, Brazil; 3 ICFO-Institut de Ciencies Fotoniques, The Barcelona Institute of Science and Technology, 08860 Castelldefels (Barcelona), Spain; 4 Max-Planck-Institut für Quantenoptik, Hans-Kopfermann-Str. 1, 85748 Garching, Germany

## Abstract

Quantum technologies promise a variety of exciting applications. Even though impressive progress has been achieved recently, a major bottleneck currently is the lack of practical certification techniques. The challenge consists of ensuring that classically intractable quantum devices perform as expected. Here we present an experimentally friendly and reliable certification tool for photonic quantum technologies: an efficient certification test for experimental preparations of multimode pure Gaussian states, pure non-Gaussian states generated by linear-optical circuits with Fock-basis states of constant boson number as inputs, and pure states generated from the latter class by post-selecting with Fock-basis measurements on ancillary modes. Only classical computing capabilities and homodyne or hetorodyne detection are required. Minimal assumptions are made on the noise or experimental capabilities of the preparation. The method constitutes a step forward in many-body quantum certification, which is ultimately about testing quantum mechanics at large scales.

## Abstract (web summary)

Photonic quantum technologies rely on the creation and manipulation of continuous variables states whose experimental preparation needs to be verified- a noteworthy impractical task. Here, the authors present a protocol that allows to certify continuous variables states with limited experimental overhead.

## Graphical abstract

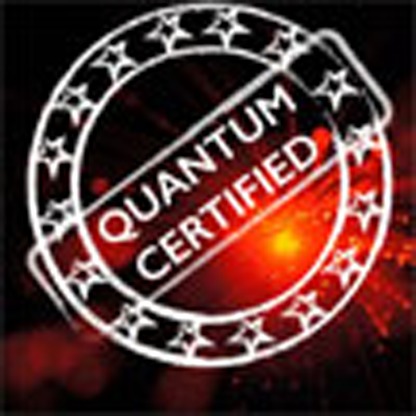

Many-body quantum devices promise exciting applications in ultraprecise quantum metrology^1^, quantum computing^2^,^3^,^4^ and quantum simulators^5^,^6^,^7^,^8^,^9^. In the quest for their large-scale realization, impressive progress on a variety of quantum technologies has recently been made^6^,^7^,^8^,^9^. Among these technologies, optical platforms play a key role. For example, sophisticated manipulations of multi-qubit entangled states of up to eight parametrically downconverted photons^10^,^11^ have been demonstrated and continuous-variable entanglement among 60 stable^12^ and up to 10,000 flying^13^ modes has been verified in optical set-ups. In addition, small-sized simulations of BosonSampling^14^,^15^,^16^,^17^ and Anderson localization in quantum walks^18^,^19^ have been performed with on-chip integrated linear-optical networks.

This fast pace of advance, however, makes the problem of reliable certification an increasingly pressing issue^20^,^21^,^22^,^23^,^24^. From a practical viewpoint, further experimental progress on many-body quantum technologies is nowadays hindered by the lack of practical certification tools. At a fundamental level, certifying many-body quantum devices is ultimately about testing quantum mechanics in regimes where it has never been tested before.

Tomographic characterization of quantum states requires the measurement of exponentially many observables. Compressed-sensing techniques^25^ reduce, for states approximated by low-rank density matrices, the requirements significantly, but still demand exponentially many measurements. Efficient certification techniques, requiring only polynomially many measurements, for universal quantum computation^26^,^27^,^28^ and a restricted model of computation with one pure qubit^29^ exist in the form of quantum interactive proofs. However, these require either a fully fledged fault-tolerant universal quantum computer^26^,^27^,^28^ or an experimentally non-trivial measurement-based quantum device^29^. In addition, these methods involve sequential interaction rounds with the device^26^,^27^,^28^,^29^. In contrast, permutationally invariant tomography^30^, tensor network techniques^31^, Monte Carlo fidelity estimation^32^,^33^,^34^, and Clifford-circuit benchmarking techniques^35^ provide experimentally friendly alternatives for the efficient certification of preparations of permutationally invariant^30^ and qubit stabilizer or W states^32^,^33^,^34^,^35^, respectively. Nevertheless, none of these methods addresses continuous-variable systems, not even in Gaussian states.

Here we introduce an experimentally friendly technique for the certification of continuous-variable state preparations without estimating the prepared state itself. First, we discuss intuitively and define precisely reliable quantum-state certification tests. We do this for two notions of certification, differing in that in one of them robustness against small preparation errors is mandatory. Then, we present a certification test, based on single-mode homodyne and heterodyne detection, for arbitrary *m*-mode pure Gaussian states, pure non-Gaussian states resulting from passive Gaussian unitary operations on Fock-basis states with *n* photons, and pure states prepared by post-selecting states in the latter class with Fock-basis measurements on *a*<*m* ancillary modes. This covers, for instance, Gaussian quantum simulations such as those of refs 12, 13 as well as the non-Gaussian ones of refs. 6, 10, 11, 14, 15, 16, 17, 18, 19. Furthermore, both photon-added or -subtracted linear-optical network states^36^,^37^,^38^,^39^ as well as all non-Gaussian states accessible to qumode-encoded qubit^40^,^41^ quantum computers also lie within the range of applicability of our method. For all Gaussian states and all mentioned non-Gaussian states with constant *n*, the protocol is efficient in *m* and, for the cases with post-selection, also in the inverse post-selection success probability.

With high probability, our test rejects all experimental preparations with a fidelity with respect to the chosen target state lower than a desired threshold and accepts if the preparation is sufficiently close to the target. That is, the protocol is robust against small preparation errors. We upper-bound the failure probability in terms of the number of experimental runs and calculate the necessary number of measurement settings. Our method is built on a fidelity lower bound, based on a natural extremality property, that is interesting in its own right. Finally, the experimental estimation of this bound relies on non-Gaussian state nullifiers, which we introduce on the way.

## Results

### Certification notions

We present our results in terms of photons propagating through optical networks, but our methods apply to any bosonic platform with equivalent dynamics. We consider a sceptic certifier, Arthur, with limited quantum capabilities, who wishes to ascertain whether an untrusted quantum prover, Merlin, presumably with more quantum capabilities, can indeed prepare certain quantum states that Arthur cannot. This mindset is reminiscent to that of quantum interactive-proof systems^26^,^27^,^28^,^29^ of computer science, but our method has the advantage that no interaction apart from the measurements of the certifier on the single-run experimental preparations from the prover is required.

In particular, we consider the situation where Merlin possesses at least a network of active single-mode squeezers and displacers as well as passive beam-splitters and phase-shifters, sufficient to efficiently implement any *m*-mode Gaussian unitary^42^,^43^,^44^,^45^,^46^, plus single-photon sources. Arthur's resources, in contrast, are restricted to classical computational power augmented with single-mode measurements. With that, he can characterize each of his single-mode measurement channels up to any desired constant precision. The task is for Merlin to provide Arthur with copies of an *m*-mode pure target state 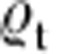 of Arthur's choice. We assume that Merlin follows independent and identical state-preparation procedures on each experimental run, described by the density matrix 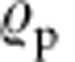. We refer to 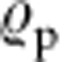 as a preparation of the target state 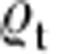. His preparation is unavoidably subject to imperfections and he might even be dishonest and try to trick Arthur. Thus, Arthur would like to run a test, with his own measurement devices, to certify whether 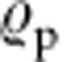 is indeed a *bona fide* preparation of 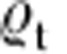.

To measure how good a preparation 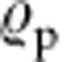 of 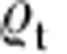 is, we use the fidelity between 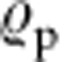 and 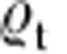, which we define as


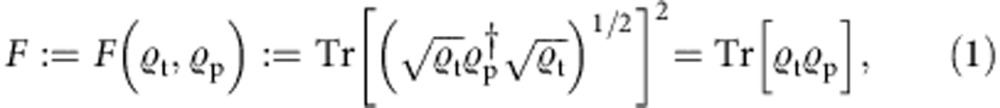


where the last equality holds because 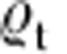 is assumed to be pure. Another usual definition of the fidelity corresponds to the square root of the fidelity as defined above. All our results can be adapted to that definition and also to the trace distance *D*:*=D*(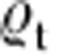, 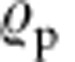), which can be defined via the 1-norm distance in state space as *D*(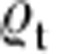,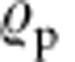):=Tr[|
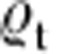
−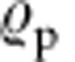
|]/2. Note that *D* can be bounded from both sides in terms of *F*, as defined in equation (1), through the well-known inequalities 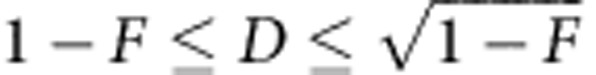, where the first inequality holds because 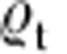 is pure.

**Figure 1:**
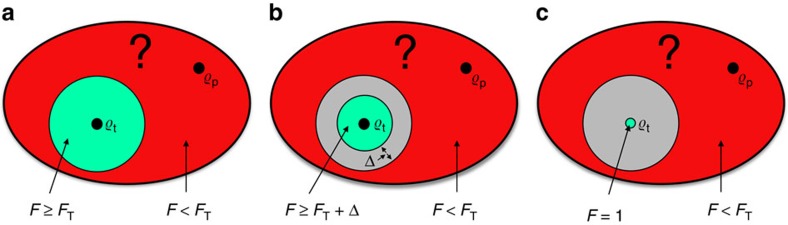
Different certification paradigms. (**a**) Naive approach: To certify an untrusted experimental preparation 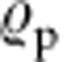 of the target state 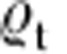, a certifier Arthur would like to run a statistical test that, for all 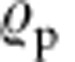, decides whether the fidelity *F* between 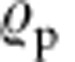 and 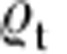 is greater or equal than a prespecified threshold *F*_T_<1 (inner green region, accept), or smaller than it (outer red region, reject). However, due to the preparations at the boundary of the two regions and experimental uncertainties, a test able to make such a decision does not exist. (**b**) The ideal scenario: A more realistic certification notion is to ask that the test rejects every 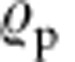 for which *F*<*F*_T_ (outer red region) and accepts every 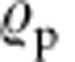 for which *F*⩾*F*_T_+Δ (inner green region), for some given Δ<1−*F*_T_. Here a buffer region of width Δ (in grey) is introduced within which the behaviour of the test can be arbitrary, but, in return, the certification is now feasible. This type of certification is thus robust against experimental infidelities as large as 1−*F*_T_−Δ. (**c**) The practical scenario: Finally, the least one can demand is that the test rejects every 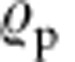 for which *F*<*F*_T_ (outer red region) and accepts at least 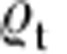 (green point). The former condition is sometimes called soundness and the latter one completeness. Here no acceptance is guaranteed for any 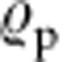 with *F*⩾*F*_T_ (grey region) other than 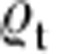 itself, but any 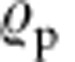 accepted by the test necessarily features *F*⩾*F*_T_. This certification notion is not necessarily robust against state deviations, but it can be more practical. In addition, in practice, the resulting tests succeed also in accepting many 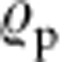
≠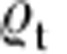 for which *F*⩾*F*_T_.

Let us first discuss what properties an experimental test must fulfil to qualify as a state certification protocol. Different certification paradigms are schematically represented in Fig. 1. We start with the formal definition of certification in the sense of Fig. 1c.

*Definition* 1 (Quantum-state certification). Let 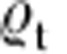 be a target state, *F*_T_<1 a threshold fidelity, and *α*>0 a maximal failure probability. A test, which takes as input copies of a preparation 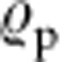 and outputs ‘accept' or ‘reject', is a certification test for 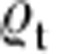 if, with probability at least 1−*α*, it both rejects every 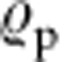 for which *F*(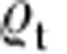,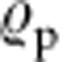) <*F*_T_ and accepts if 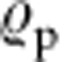
=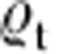. We say that any 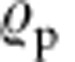 accepted by such a test is a certified preparation of 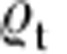.

### Classes of target states

To specify the target states we need to introduce some notation. We denote *m*-mode Fock basis states by 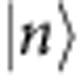, with **n**:=(*n*_1_, *n*_2_,…,*n*_*m*_) being the sequence of photon numbers *n*_*j*_⩾0 in each mode *j*∈[*m*], where the short-hand notation [*m*]:={1, 2,…,*m*} is introduced, and call 
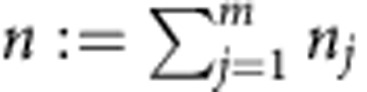
 the total input photon number. In particular, we will pay special attention to Fock basis states 
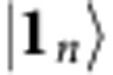
 with exactly one photon in each of the first *n* modes and the vacuum in the remaining *m*−*n* ones, that is, those for which **n**=**1**_*n*_, with


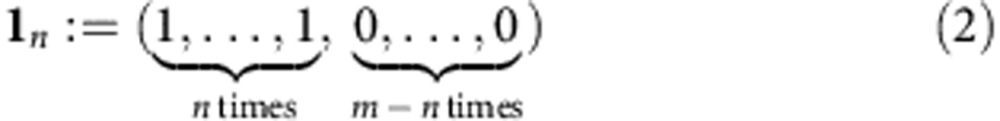


Note that 
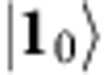
 is the Gaussian vacuum state 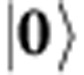. We denote the photon number operator corresponding to mode *j* by 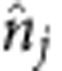 and the total photon number operator by 
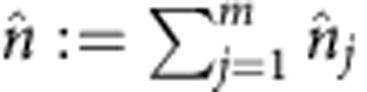
.

In addition, for post-selected target states, we denote by 
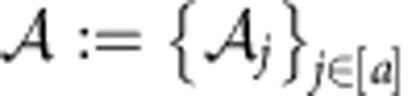
, where each element 
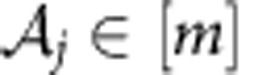
 labels a different mode, the subset of 
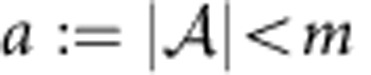
 modes on which the post-selection measurements are made. We then identify the remaining *m*−*a* modes as the system subset 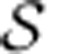, which carries the post-selected target state 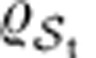. The subindex 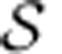 emphasizes that 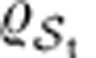 represents an (*m*−*a*)-mode post-selected target state and distinguishes it from *m*-mode target states without post-selection, which we denote simply as 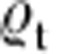. We denote by 
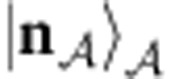
, with 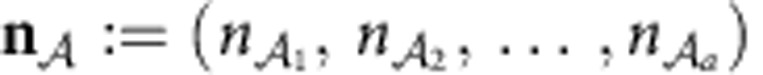, an *a*-mode pure normalized Fock-basis state of 
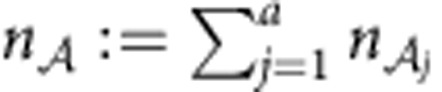
 total photons on the modes 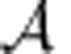. We use the short-hand notations 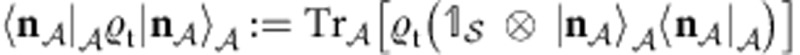, where 
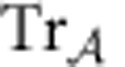
 indicates partial trace over the Fock space of 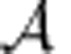, 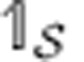 denotes the identity on 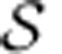, and 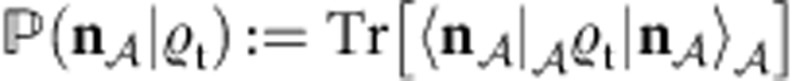 is the post-selection success probability, that is, the probability of measuring 
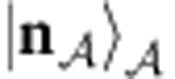
 in a projective measurement on 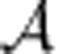. Without loss of generality, we consider throughout only the non-trivial case 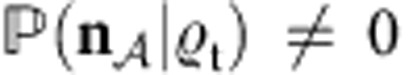.

With the notation introduced, we derive our results for: arbitrary *m*-mode pure Gaussian states, given by the class


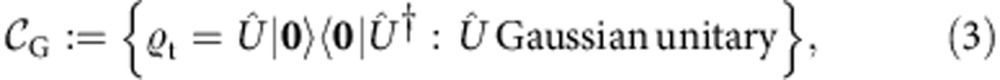


*m*-mode pure linear-optical network states from the class


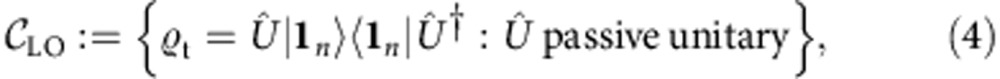


and (*m*−*a*)-mode pure locally post-selected linear-optical network states from the class


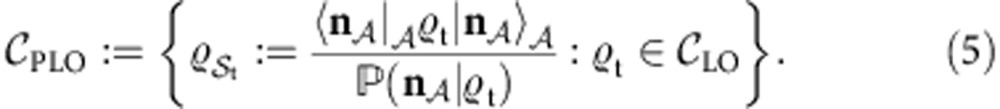


**Figure 2:**
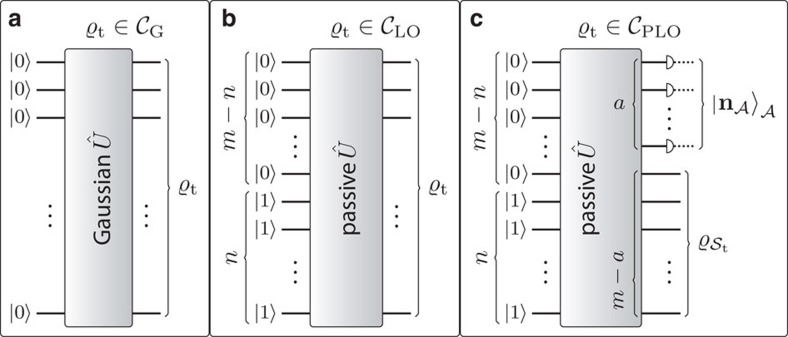
Classes of target states. (**a**) 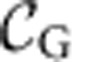 is the class composed of all *m*-mode pure Gaussian states. These can be prepared by applying an arbitrary Gaussian unitary *Û* (possibly involving multimode squeezing) to the *m*-mode vacuum state 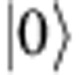. (**b**) The class 
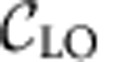
 includes all *m*-mode pure non-Gaussian states produced at the output of an arbitrary linear-optical network, which implements a passive Gaussian unitary *Û* (without squeezing), with the Fock-basis state 
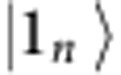
 containing one photon in each of the first *n* modes and zero in the remaining *m*−*n* ones as input. As the order of the modes is arbitrary, choosing the first *n* modes as the populated ones does not constitute a restriction. (**c**) The third class, 
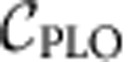
 encompasses all (*m*−*a*)-mode pure non-Gaussian states obtained by projecting a subset 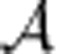 of *a*<*m* modes of an *m*-mode pure linear-optical network state 
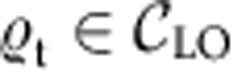
 onto a pure normalized product Fock-basis state 
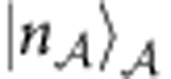
. In practice, this is done probabilistically by measuring 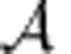 in a local basis that contains 
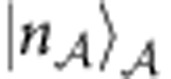
 and post-selecting only the events in which 
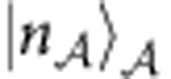
 is measured. Thus, the *a* modes in 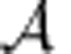 are used as ancillas, whereas the effective system is given by the subset 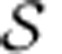 containing the other *m*−*a* modes, which carries the final target state. For concreteness, but without any loss of generality, in the plot, the ancillary modes are chosen to be the last *a* ones. These three classes cover the target states considered in the vast majority of quantum photonic experiments.

The three classes of target states are schematically represented in Fig. 2. The class 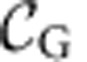 is crucial within the realm of ‘continuous-variable' quantum optics and quantum information processing. It encompasses, for instance, ‘twin-beam' (two-mode squeezed vacuum) states under passive networks, which are used to simulate, upon coincidence detection, multi-qubit states^6^. The class 
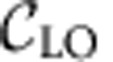
 includes all the settings sometimes referred to as ‘discrete variable' linear-optical networks. This class covers, among others, the targets of several recent experimental simulations with on-chip integrated linear-optical networks^14^,^15^,^16^,^17^,^18^,^19^. The third class, 
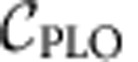
, is the one of linear-optical network states locally post-selected with Fock-basis measurements. This class includes important non-Gaussian resources for quantum information and quantum optics. For instance, it encompasses both photon-added or -subtracted linear-optical network states^36^,^37^,^38^,^39^. Furthermore, when *n* is proportional to *m*, it also includes all the states prepared by probabilistic schemes of the type of refs 40, 41 for universal qumode-encoded qubit quantum computation.

### The certification test

The basis of the our certification scheme is a technique for the estimation of the quantity


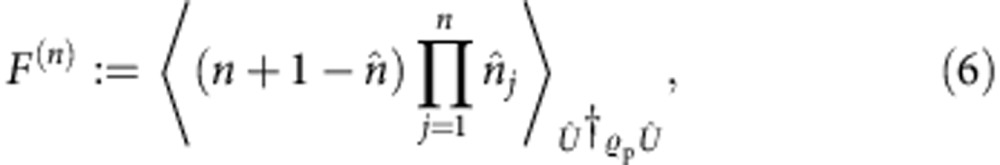


with *n* the total input photon number. As shown in the Methods section, for all target states 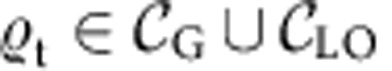, *F*^(*n*)^ is a lower bound on the fidelity *F* and, moreover, *F*^(*n*)^=*F*=1 if 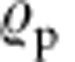
=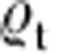. In addition, this bound is connected to a natural extremality property of Gaussian states, discussed also in the Methods section. Our test 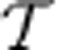, summarized in Box 1, yields an estimate *F*^(*n*)*^ of *F*^(*n*)^. If *F*^(*n*)*^ is sufficiently above the threshold *F*_T_, the preparation 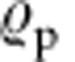 is accepted. Otherwise it is rejected. We introduce the measurement schemes 
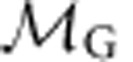
 and 
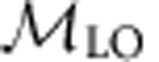
, which depend on the specific target state, to obtain the estimate *F*^(*n*)*^. Gaussian states can be estimated with the scheme 
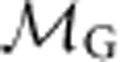
, while linear-optical network states with 
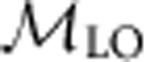
. Both measurement schemes are summarized in the Methods section and described in detail in Supplementary Note 1. In turn, a fidelity bound for post-selected target states in 
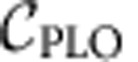
 similar to *F*^(*n*)^ is presented in the Methods section. Its derivation, the adaptation of the test 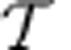 to post-selected targets, and the corresponding measurement scheme are provided in Supplementary Note 2.

Our theorems guarantee that the test from Box 1 is indeed a certification test and give a bound on the scaling of the number of samples that are needed for the test. To state them, we introduce some notation related to mode space descriptions of linear-optical networks first. Any Gaussian unitary transformation 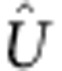 on Hilbert space can be represented by

an affine symplectic transformation in mode space, that is, by a symplectic matrix 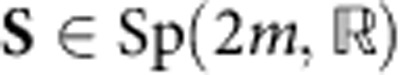 followed by a phase-space displacement 
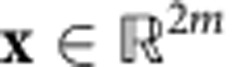
 (see equation (25) in the Methods section), where the real-symplectic group 
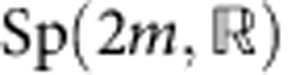
 contains all real 2*m* × 2*m* matrices that preserve the canonical phase-space commutation relations^42^,^43^. By virtue of the Euler decomposition^42^,^45^, **S** can be implemented with single-mode squeezing operations and passive mode transformations. We denote the maximum single-mode squeezing of **S** by *s*_max_ and define the mode range *d*≤*m* to be the maximal number of input modes to which each output mode is coupled (for details see Supplementary Note 1). Also, it will be useful to define


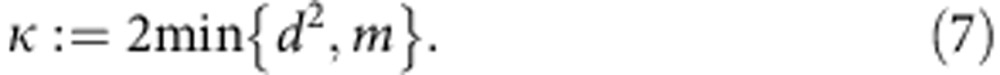


The displacement **x** can be implemented by a single-mode displacer at each mode *j*∈[*m*], with amplitude (*x*_2*j*−1_, *x*_2*j*_), where *x*_*k*_, for *k*∈[2*m*], is the *k*th component of **x**. The vector 2-norm is denoted by 
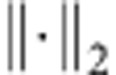
, that is, 
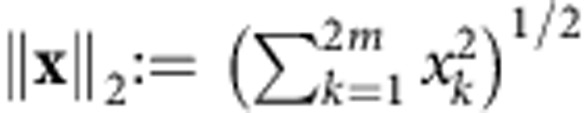
.

We take *σ*_*i*_ to be a uniform upper bound on the variances of any product of *i* phase-space quadratures in the state 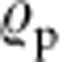. If 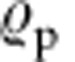 is Gaussian, then *σ*_1_ and *σ*_2_ are functions of the single-mode squeezing parameters of 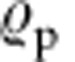. In addition, we call 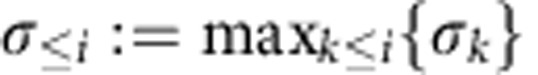 the maximal *i*th variance of 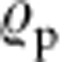. Finally, we use the Landau symbol O to denote asymptotic upper bounds.

*Theorem* 2 (Quantum certification of Gaussian states). Let *F*_T_<1 be a threshold fidelity, α>0 a maximal failure probability, and 0<*ɛ*≤(1−*F*_T_)/2 an estimation error. Let 
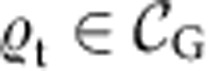
 have maximum single-mode squeezing *s*_max_⩾1, mode range *d*≤*m*, and displacement **x**. Test 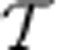 from Box 1 is a certification test for 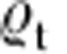 and requires at most


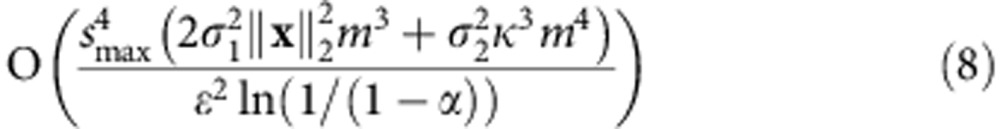


copies of a preparation 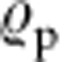 with first and second variance bounds *σ*_1_>0 and *σ*_2_>0, respectively.

*Theorem* 3 (Quantum certification of linear-optical network states). Let *F*_T_<1 be a threshold fidelity, *α*>0 a maximal failure probability, and 0<*ɛ* ≤(1−*F*_T_)/2 an estimation error. Let 
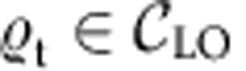
 have mode range *d* ≤*m*. Test 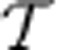 from Box 1 is a certification test for 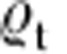 and requires at most


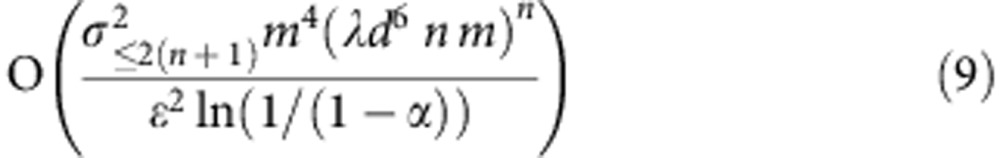


copies of a preparation 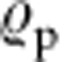 with maximal 2(*n*+1)-th variance *σ*_≤2(*n*+1)_, where *λ*>0 is an absolute constant.

The proofs of all our theorems are provided in the Supplementary Information. The treatment of the class 
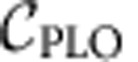
 follows as a corollary of Theorem 3 and is also provided in Supplementary Note 2. Equations (8) and (9) are highly simplified upper bounds on the total number of copies of 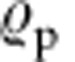 that 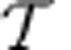 requires. For more precise expressions see Supplementary Lemmas 6 and 9. Note that neither of the two theorems requires any energy cut-off or phase-space truncation. While the bound in equation (9) is inefficient in *n*, both for the Gaussian and linear-optical cases, the number of copies of 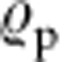 scales polynomially with all other parameters, in particular with *m*. Thus, arbitrary *m*-mode target states from the classes 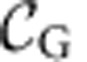 and 
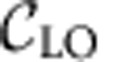
 with constant *n*, are certified by 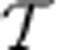 efficiently.

Interestingly, since states in 
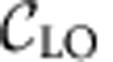
 in general display negative Wigner functions, sampling from their measurement probability distributions cannot be efficiently done by the available classical sampling methods^47^,^48^,^49^. Furthermore, for Fock-state measurements, these distributions define BosonSampling, for which hardness results exist^50^ for *m* asymptotically lower bounded by *n*^5^.

Also, note that there are no restrictions on 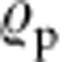 except that, in practice, to apply the theorems, one needs bounds on *σ*_1_, *σ*_2_, and *σ*_≤2(*n*+1)_. These variances are properties of 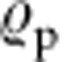 and are therefore *a priori* unknown to Arthur. However, he can reasonably estimate them from his measurements. Note that, for random variables that can take any real value, assuming that the variances are bounded is a fundamental and unavoidable assumption to make estimations from samples; and it is one that can be contrasted with the measurement results.

### Robustness against preparation imperfections

To end up with, we consider certification in the sense of Fig. 1b:

*Definition* 4 (Robust quantum-state certification). Let 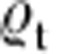 be a target state, *F*_T_<1 be a threshold fidelity, *α*>0 a maximal failure probability, and Δ<1−*F*_T_ a fidelity gap. A test, which takes as input copies of a preparation 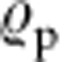 and outputs ‘accept' or ‘reject', is a robust certification test for 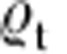 if, with probability at least 1−α, it both rejects every 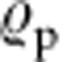 for which *F*(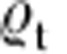, 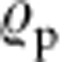)<*F*_T_ and accepts every 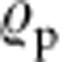 for which *F*(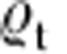, 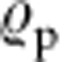)⩾*F*_T_+Δ. We say that any 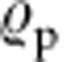 accepted by such a test is a certified preparation of 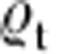.

This definition is more stringent than Definition 1 in that it guarantees that preparations sufficiently close to 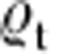 are necessarily accepted, rendering the certification robust against preparation imperfections causing fidelity deviations as large as 1−(*F*_T_+Δ). We now show that our test 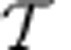 from Box 1 is actually a robust certification test.

To this end, we first write 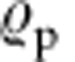 as


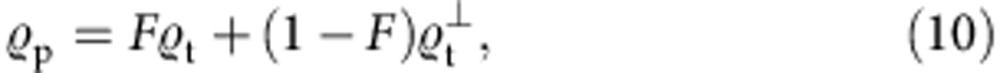


where 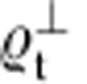 is an operator orthogonal to 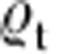 with respect to the Hilbert–Schmidt inner product, that is, such that 
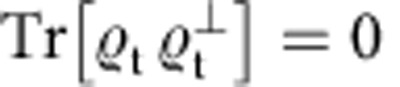
. As 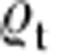 is assumed to be pure, it follows immediately that 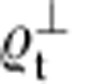 is actually a state. In fact, multiplying both sides of equation (10) by 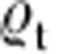 and taking the trace, one readily sees that decomposition in equation (10) is just another way to express the fidelity in equation (1).

According to equation (6), the lower bound *F*^(*n*)^ can be defined as an expectation value of the observable


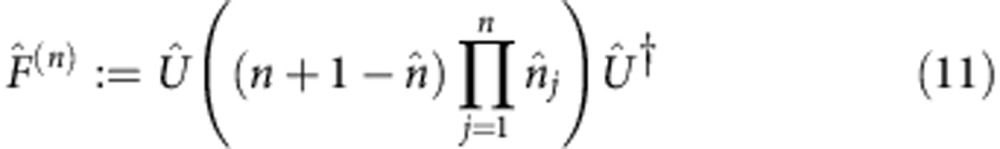


with respect to 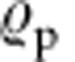. In a similar way, we define the quantity


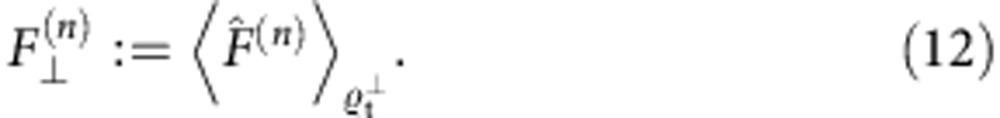


By taking the expectation value of equation (10) with respect to the observable 
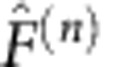
 and using that 
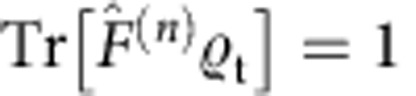
 and *F*^(*n*)^≤*F*, one finds that 
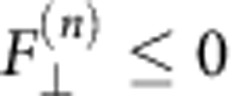
. The parameter 
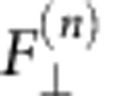
 turns out to quantify the robustness of our certification test.

*Theorem* 5 (Robust quantum certification). Under the same conditions as in Theorems 2 and 3, test 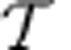 from Box 1 is a robust certification test with fidelity gap


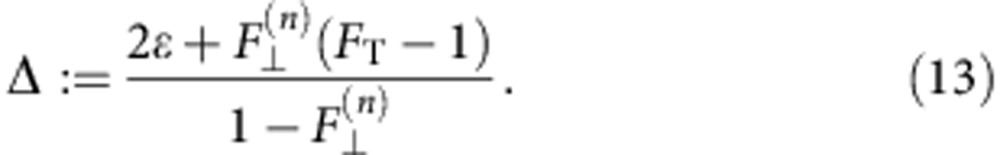


Since 
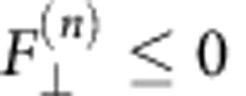
 and *F*_T_<1, it is clear that Δ>0. On the other hand, note that 
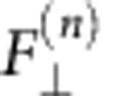
 can in general be arbitrarily smaller than zero. This happens, for instance, for preparations for which 
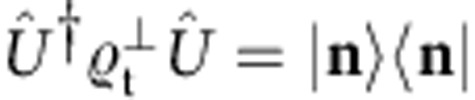
, with *n*_1_, *n*_2_,…,*n*_*n*_⩾1 and *n* arbitrarily large. In particular, in the limit 
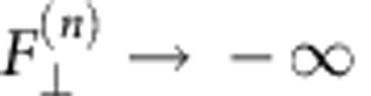
, it holds that Δ→1−*F*_T_, so that the certification becomes less robust with decreasing 
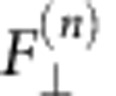
, as one would expect. In contrast, as 
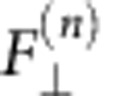
 increases from −∞ to 0, the gap decreases to its minimal value Δ=2*ɛ*. Note that, since it depends on 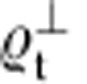, 
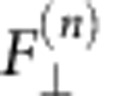
 cannot be directly estimated from measurements on 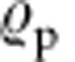 alone. However, Theorem 5 guarantees the existence of an entire closed convex set of states around 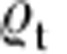 that are rightfully accepted and Δ lower bounds the size of that region. Furthermore, in experimentally relevant situations, 
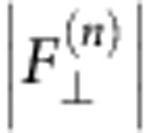
 is expected to be small, meaning that Δ is close to its optimal value 2*ɛ*.

Finally, a statement equivalent to Theorem 5 for target states 
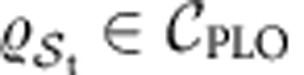
 follows as an immediate corollary of it and is Supplementary Note 2.

## Discussion

Large-scale photonic quantum technologies promise important scientific advances and technological applications. So far, considerably more effort has been put into their realization than into the verification of their correct functioning and reliability. This imposes a serious obstacle for further experimental advance, specifically in the light of the speed at which progress towards many-mode architectures takes place. Here we have presented a practical reliable certification tool for a broad family of multimode bosonic quantum technologies.

We have proven theorems that upper bound the number of experimental runs sufficient for our protocol to be a certification test. Our theorems provide large-deviation bounds from a simple extremality-based fidelity lower bound that is interesting in its own right. Our theorems hold only for statistical errors, but the stability analysis on which they rely (see Supplementary Lemmas 5 and 8) holds regardless of the nature of the errors. In Supplementary Note 5, we show that our fidelity estimates are robust also against small systematic errors.

From a more practical viewpoint, our test allows one to certify the state preparations of most current optical experiments, in both the ‘continuous-variable' and the ‘discrete-variable' settings. This is achieved under the minimal possible assumptions: namely, only that the variances of the measurement outcomes are finite. Thus, the certification is as unconditional as the fundamental laws of statistics allow. In particular, no assumption on the type of noise is made. Despite the rigorous bounds on the estimation errors and failure probabilities, our methods are both experimentally friendly and resource efficient.

Notably, our test can efficiently certify multimode negative-Wigner-function states that define, via local measurements, sampling problems whose classical simulation is not known to be efficient^47^,^48^,^49^. For instance, it can be applied to the certification of optical circuits of the type used in BosonSampling: There, *m*-mode Fock-basis states of *n* photons are subjected to a linear-optical network described by a random unitary *Û* drawn from the Haar measure^50^ and, subsequently, each output mode is measured in the Fock basis. While the question of the certification of the classical outcomes of such samplers without assumptions on the device is still largely open^20^,^21^, with the methods described here the premeasurement non-Gaussian quantum outputs of BosonSampling devices^14^,^15^,^16^,^17^ can be certified reliably and, for constant *n*, even efficiently. In this sense, this work goes significantly beyond previously proposed schemes to rule out particular cheating strategies by the prover^21^,^22^,^23^,^24^. Furthermore, a variety of non-Gaussian states paradigmatic in quantum optics and quantum information are also covered by our protocol (see Supplementary Note 2 for details). These include, for instance, linear-optical network outputs post-selected though photon-number measurements, ranging from both photon-added or -subtracted linear-optical network states^36^,^37^,^38^,^39^ to all the states preparable with Knill–Laflamme–Milburn-like schemes^40^,^41^. For all such states, our test is efficient in the inverse post-selection success probability 
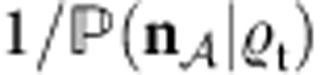
.

The present method constitutes a step forward in the field of photonic quantum certification, with potential implications on the certification of other many-body quantum-information technologies. Apart from that of BosonSamplers and optical schemes with post-selection, the efficient and reliable certification of large-scale photonic networks as those used, for instance, for multimode Gaussian quantum-information processing^12^,^13^, non-Gaussian Anderson-localization simulations^18^,^19^, and quantum metrology^1^, with a constant number of input photons, is now within reach.

## Methods

### Fidelity lower bound

Here we formalize the extremality notion and derive a lower bound on the fidelity *F* for non post-selected target states. All the non post-selected target states we consider are of the form


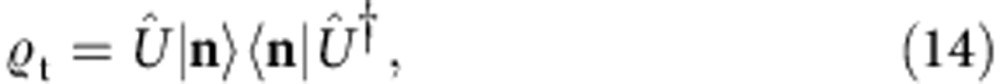


where 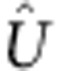 is an arbitrary Gaussian unitary and 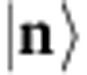 an arbitrary Fock-basis state. First, we derive a fidelity lower bound for general states of the form given in equation (14) and then consider the linear-optical and Gaussian cases separately. Lower bounds for the post-selected target states are provided further below in the Measurement Scheme.

We start recalling that


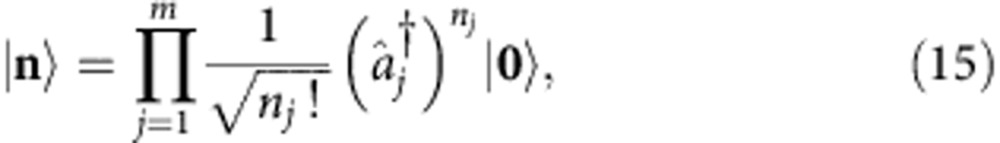


where 
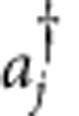
 is the creation operator of the *j*th mode. Its Hermitian conjugated 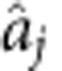 is the corresponding annihilation operator. These operators satisfy 
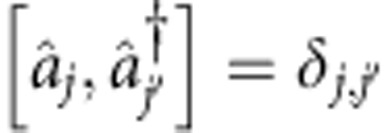
, where *δ*_*j*,*j*′_ denotes the Kronecker delta of *j* and *j*′, and 
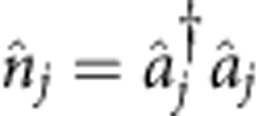
, for all *j*, *j*′∈[*m*]. The fidelity in equation (1) can be written as 
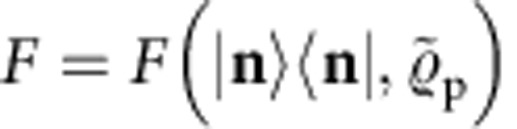
, where 
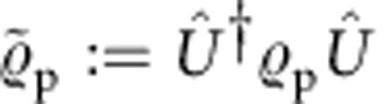
 is the Heisenberg representation of 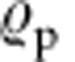 with respect to 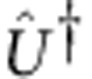. With this, equation (15), and the cyclicality property of the trace, we obtain that


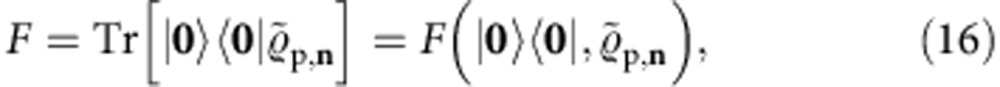


where


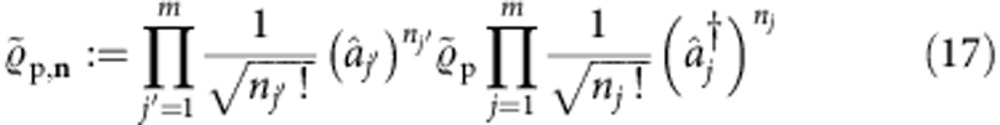


is a (not necessarily normalized) positive-semidefinite operator.

To lower bound 
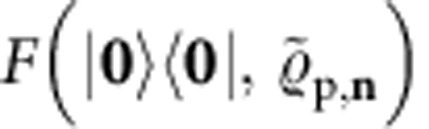
, we consider the expectation value 
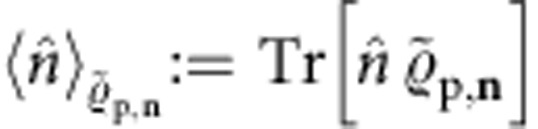
. We write for the identity operator. From the facts 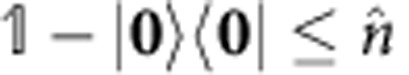 and 
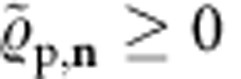
, it follows that


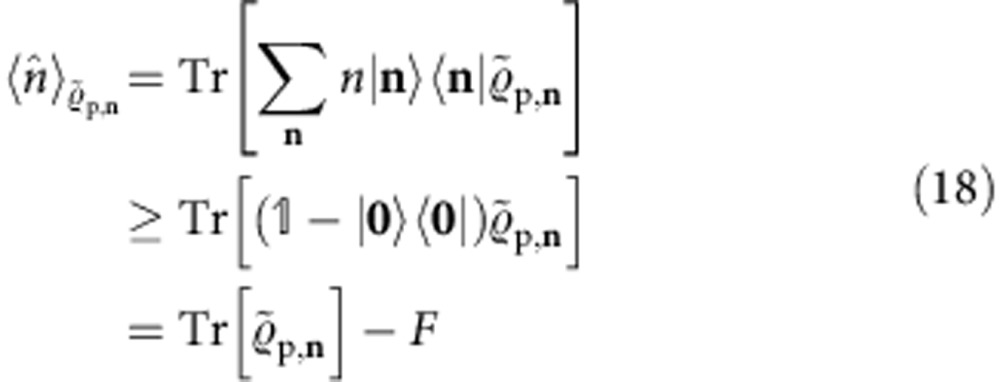


and hence,


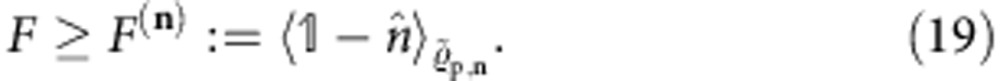


For 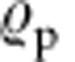
=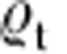 it holds that 
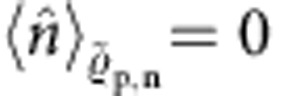
 and 
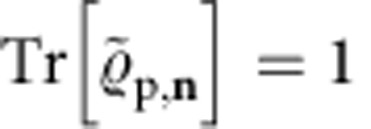
. Thus, for 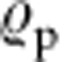
=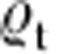 the inequality in equation (18) becomes an equality and, therefore, the bound in equation (19) is then saturated, as announced earlier.

Next, we define the operator valued Pochhammer-symbol


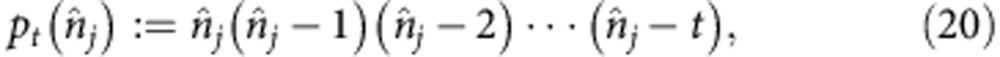


for any integer *t*⩾0, and *p*_−1_(*x*):=1. In Supplementary Note 6 we show that


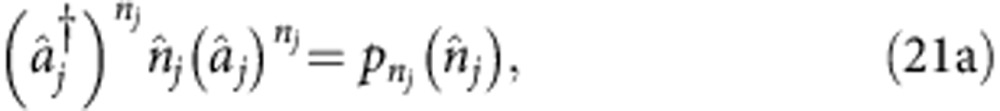


and


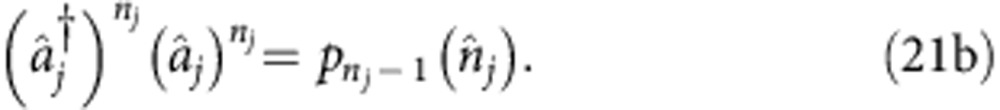


Inserting equation (17) into equation (19), using the cyclicity property of the trace, grouping the operators of each mode together, using equation (21a) and equation (21b), and that 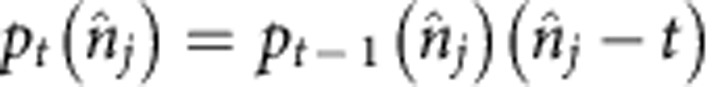, we obtain the general fidelity lower bound


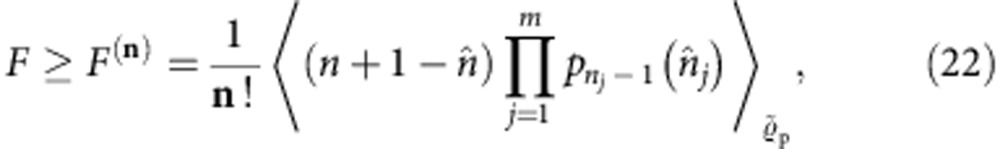


where **n**! :=*n*_1_!*n*_2_!…*n*_*m*_!.

In order to specialize to the linear-optical case 
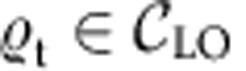
, we take **n**=**1**_*n*_, i.e., *n*_*j*_=1 for all *j*∈[*n*] and *n*_*j*_=0 otherwise. With this, *F*^(**n**)^ in equation (22) simplifies to the bound *F*^(*n*)^ in equation (6). Finally, to restrict it to the Gaussian case 
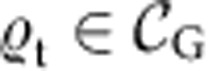
, we take *n*_*j*_=0 for all *j*∈[*m*]. This yields the particularly simple expression


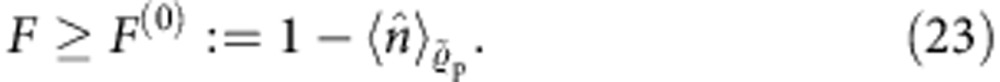


The last expression manifests the above-mentioned connection between our fidelity lower bound and an intuitive extremality property of Gaussian states. Namely, the lower the average number of photons of 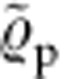 is, the closer to the vacuum it must be and, therefore, the closer 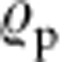 to the target state 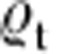.

Arthur does not have, in general, enough quantum capabilities to directly estimate 
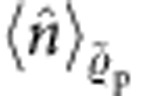
 by undoing the operation *Û* on Merlin's outputs and then measuring 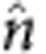 in the Fock-state basis. However, we show in the next section that he can efficiently obtain 
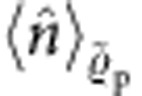
, as well as the expectation values in equations (22) and (6), from the results of single-mode homodyne or heterodyne measurements.

### Measurement scheme

First, we introduce some notation. By 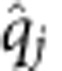 and 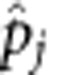 we denote, respectively, the conjugated position and momentum phase-space quadrature operators of the *j*th mode in the canonical convention^42^,^43^, that is, with the commutation relations 
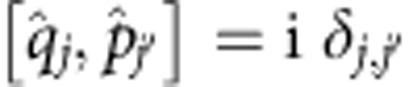
. The particle number operator of the *j*th mode can be written in terms of the phase-space quadratures as 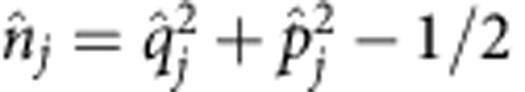. In addition, it will be convenient to group all quadrature operators into a 2*m*-component column vector 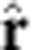, with elements


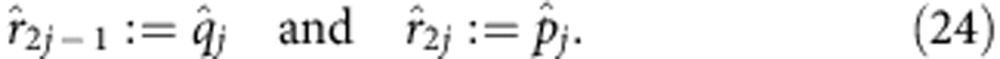


As already mentioned, the action of *Û* on mode space is given by a symplectic matrix 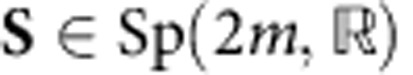 and a displacement vector 
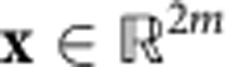
. More precisely, under a Gaussian unitary *Û*, 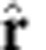 transforms according to the affine linear map^42^


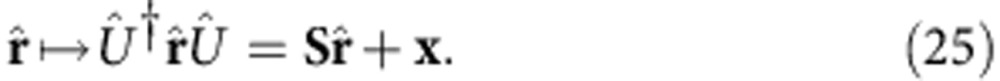


Equivalently, the right-hand side of this equation defines the Heisenberg representation of 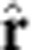 with respect to *Û*. In addition, it will be useful to denote the Heisenberg representation of 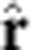 with respect to *Û*^†^ by 
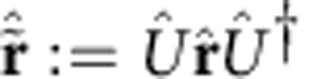
. Thanks to equation (25), we can write 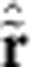 in terms of the symplectic matrix **S** and displacement vector **x** that define *Û*, as


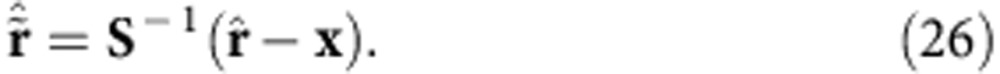


The symbols 
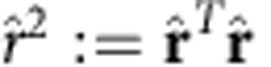
 and 
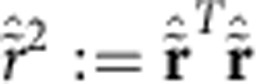
 will represent, respectively, the scalar products of 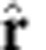 and 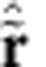 with themselves. Also, we will use the same notation for the Heisenberg representations of each quadrature operator with respect to *Û*^†^, that is, 
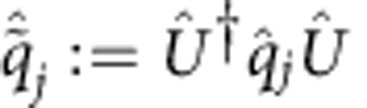
 and 
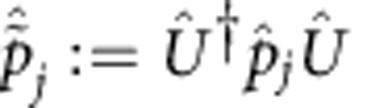
.

Next, for *β*∈{0, *n*, **n**}, we express our fidelity bounds in the general form


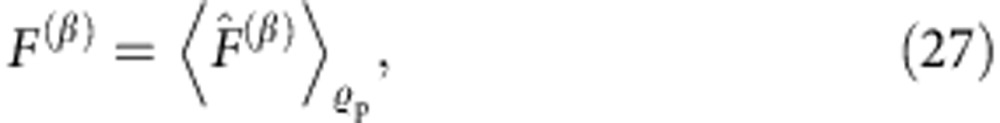


where 
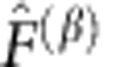
 is an observable decomposed explicitly in terms of the local observables to which Arthur has access. We start with the Gaussian case 
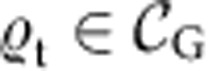
. To express the bound of equation (23) as in equation (27), we first write the total photon-number operator as


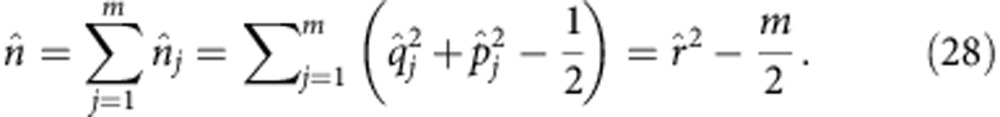


This, in combination with equation (23), yields


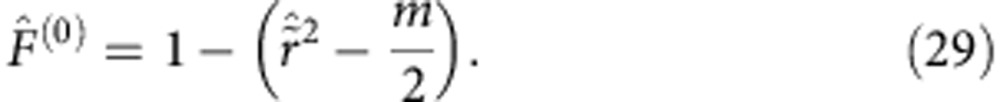


Note that, due to equation (26), each component of 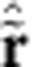 is a linear combination of at most 2*m* components of 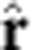. This implies that Arthur can obtain 
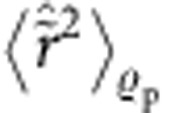
 by measuring at most 2*m* single-quadrature expectation values of the form 
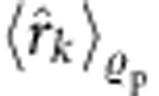
 and 4*m*^2^ second moments of the form 
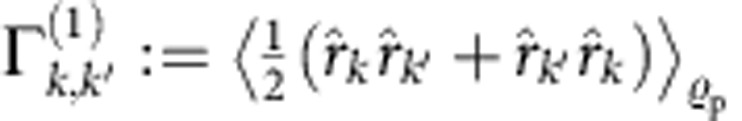
. He can then classically efficiently combine them as dictated by **S** and **x** in equation (26). In Supplementary Note 1, we give the details of this measurement procedure, which we call 
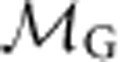
, and show that measuring *mκ* second moments, instead of 4*m*^2^, is actually enough. Furthermore, in Supplementary Note 4, we show that only *m*+3 experimental settings suffice if homodyne detection is used and a single setting if heterodyne detection is used.

Now, proceeding in a similar manner with the generic bound of equation (22), we obtain


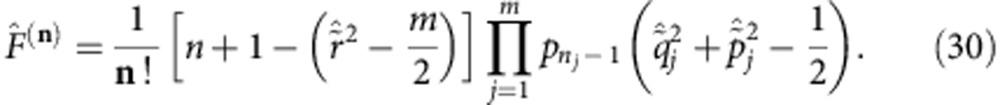


Note that the observable in equation (29) is contained as the special case *n*=0. For target states in the class 
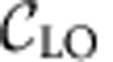
, *Û* is assumed to be a passive Gaussian unitary. Such unitaries preserve the area in phase-space, that is, if 
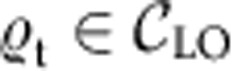
 it holds that 
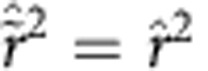
 (for details, see Supplementary Note 1). Hence, using this and specialising to the case **n**=**1**_*n*_, equation (30) simplifies to


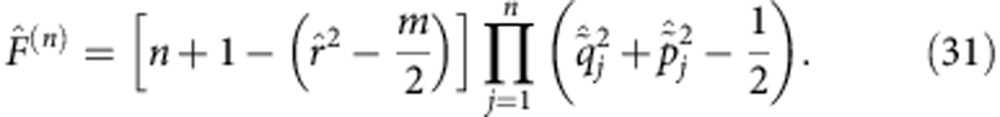


Again by virtue of equation (26), Arthur can now obtain the expectation values of the observables in equations (30) and (31) by measuring 2*j*th moments of the form 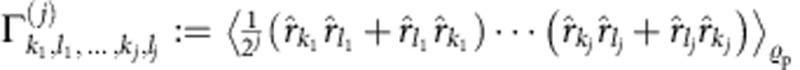 and then classically recombining them, which—for constant *n*—he ca*n* do efficiently. In Supplementary Note 1, we give the details of the measurement procedure to obtain *F*^(*n*)^, which we call 
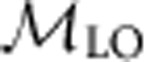
. In particular, we show that, to obtain 
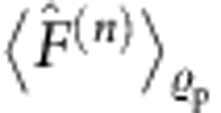
, estimating a total of O(*m*(4*d*^2^+1)^*n*^) 2*j*th moments, with *j*∈[*n*+1], is enough. Also, we list which moments are the relevant ones in terms of 
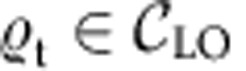
. Furthermore, in Supplementary Note 4, we show that a single heterodyne experimental setting throughout suffices here too.

Finally, in Supplementary Note 2, we derive a bound analogous to that of equations (27) and (31) for post-selected target states 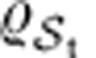. More precisely, we show that the fidelity 
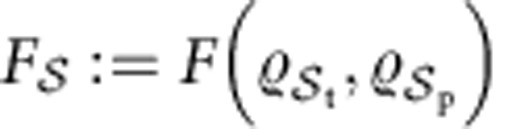
 between 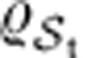 and an arbitrary, unknown (*m*−*a*)-mode system preparation 
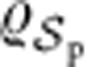
 is lower bounded as


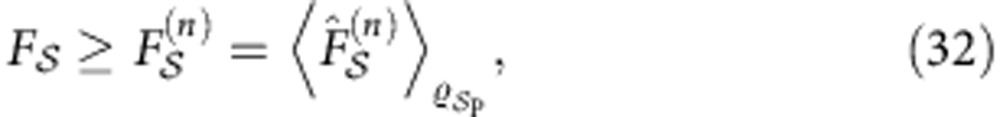



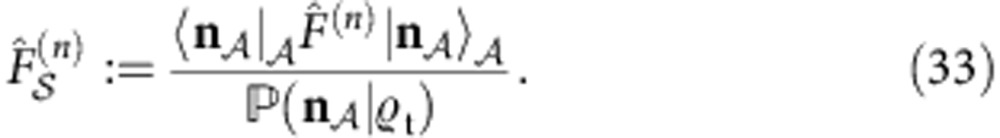


Actually, the bound holds not only for target states 
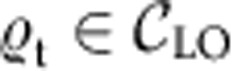
 projected onto 
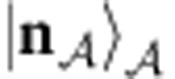
 but also for the more general target states of equation (14), with *Û* any Gaussian unitary and 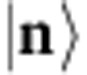 any Fock-basis state, projected onto any generic *a*-mode pure product state on 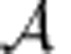. Apart from being experimentally more relevant, linear-optical network target states post-selected with Fock-basis measurements possess the peculiarity that the corresponding bound is tight for perfect preparations. That is, for these states, if 
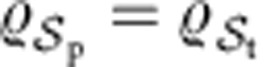
 then 
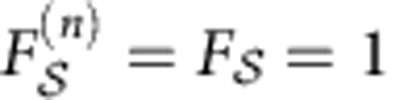
, just as in the cases without post-selection.

### Non-Gaussian state nullifiers

It is instructive to mention that the observables


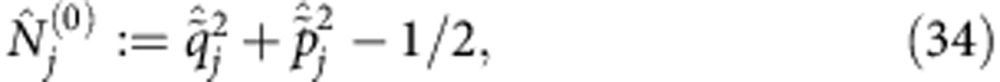


for *j*∈[*m*], correspond to the so-called nullifiers of the Gaussian states in 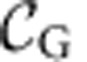. The nullifiers are commuting operators that, despite originally introduced^51^ as a tool to define Gaussian graph states, can be tailored to define any pure Gaussian state^52^,^53^: If a state is the simultaneous null-eigenvalue eigenstate of all *m* nullifiers of a given pure Gaussian state, then the former is necessarily equal to the latter. The bound *F*^(0)^, given by equations (27) and (29), exploits the fact that if a preparation gives a sufficiently low expectation value for the sum 
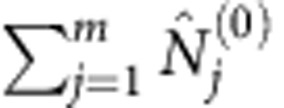
 of all *m* nullifiers, then its fidelity with the target state must be high. A similar intuition has been previously exploited^12^,^13^ to experimentally check for multimode entanglement of ultralarge Gaussian cluster states. Here we cannot only certify entanglement but the quantum state itself.

Analogously, in the non-Gaussian case, from the derivation of equation (30) and the fact that


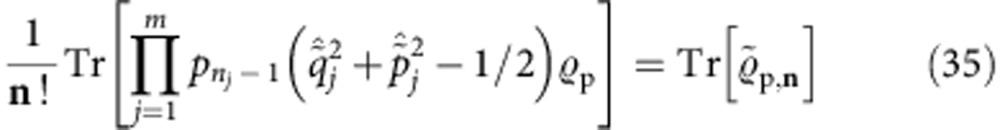


equals 1 for 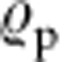
=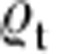, we can identify the observable


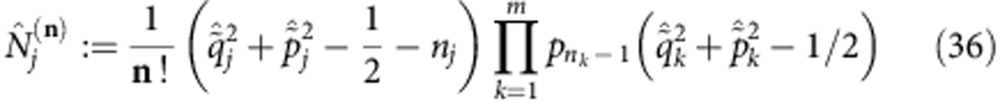


as the *j*th nullifier of the *m*-mode non-Gaussian state 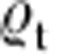 of equation (14). Indeed, all *m* observables given by equation (36) for all *j*∈[*m*] commute and have 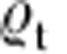 as their unique, simultaneous null-eigenvalue eigenstate. To end up with, due to the projection onto 
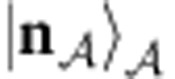
, the equivalent observables for 
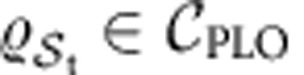
 do not in general commute. Nevertheless, their linear combination given by 
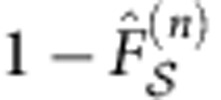
 still defines an observable with 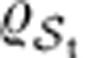 as its unique null-eigenvalue eigenstate. These observables constitute, to our knowledge^42^,^52^,^53^, the first examples of nullifiers for non-Gaussian states.

## Additional information

**How to cite this article:** Aolita, L. *et al*. Reliable quantum certification of photonic state preparations. *Nat. Commun.* 6:8498 doi: 10.1038/ncomms9498 (2015).

## Supplementary Material

Supplementary Information Supplementary Notes 1-6 and Supplementary References

## (Certification test ).

**Settings adjustments:** Arthur chooses a threshold fidelity *F*_T_<1, a maximal failure probability *α*>0, and an estimation error 0<*ɛ*≤(1−*F*_T_)/2.

**State request:** Arthur provides Merlin with the classical specification *n*, **S** and **x** of the target state  and requests a sufficient number of copies of it.

**Quantum measurements:** If *n*=0, Arthur measures 2*mκ* two-mode correlations and 2*m* single-mode expectation values specified by the measurement scheme  (see the Methods section), which can be done with *m*+3 local homodyne settings or a single local heterodyne setting throughout.

If *n*>0, he measures O(*m*(4*d*^2^+1)^*n*^) *m*ulti-body correlators, each one involving between 1 and 2*n*+1 modes, specified by the measurement scheme  (see the Methods section), which can be done with a single local heterodyne setting throughout.

**Classical post-processing:** By processing the measurement outcomes (see the Methods section), he obtains a fidelity estimate *F*^(*n*)*^ such that *F*^(*n*)*^∈[*F*^(*n*)^−ɛ, *F*^(*n*)^+*ɛ*] with probability at least 1−*α*, where *F*^(*n*)^ is the lower bound to *F* given by expression (6).

**Accept-reject decision:** If *F*^(*n*)*^<*F*_T_+*ɛ*, he rejects. Otherwise, he accepts.
