# Supplementary material for: Reliable quantum certification of photonic state preparations
Source: Nat Commun. 2015 Nov 18;6:8498. doi: 10.1038/ncomms9498 (PMC4673657; doi:10.1038/ncomms9498)
Supplement: Supplementary Information — Supplementary Notes 1-6 and Supplementary References [file ncomms9498-s1.pdf]

Supplementary Information

Supplementary Note 1 - The measurement scheme.

In this section we elaborate on the fidelity bounds $F^{(0)}$ and $F^{(n)}$ of the fidelity bounds for the Gaussian and linear-optical case, respectively. To this end, it will be convenient to first specify some details of the symplectic matrix $\mathbf{S}$, which describes the optical network.

By virtue of the Euler decomposition[6,7], $\mathbf{S}$ can be decomposed as

$$\mathbf{S} = \mathbf{O}\,\mathbf{D}\,\mathbf{O}', \tag{1}$$

where $\mathbf{D} \in \mathbb{R}^{2m\times 2m}$ is positive-definite and diagonal, with elements $D_{2j-1,2j-1} \coloneqq s_j \geq 1$ and $D_{2j,2j} \coloneqq s_j^{-1}$, for $j \in [m]$, and $\mathbf{O} \in \mathbb{R}^{2m\times 2m}$ and $\mathbf{O}' \in \mathbb{R}^{2m\times 2m}$ are orthogonal matrices. $\mathbf{D}$ describes m active single-mode squeezers in parallel, each one with squeezing parameter s_j along the position quadrature. The maximum single-mode squeezing is $s_{\max} \coloneqq \max_{1\leq j\leq m}\{s_j\}$. $\mathbf{O}$ and $\mathbf{O}'$, in turn, describe passive mode transformations that can be implemented by linear-optical networks of at most $m(m-1)/2$ beam-splitters and single-mode phase shifters[8]. In the two settings considered here, i.e., for any $\varrho_{\mathrm{t}} \in \mathcal{C}_{\mathrm{G}} \cup \mathcal{C}_{\mathrm{LO}}$, the unitary $\hat{U}$ in equations (3) and (4) is such that $\mathbf{O}'$ can be taken as the identity matrix. In the first setting, i.e., for $\varrho_{\mathrm{t}} \in \mathcal{C}_{\mathrm{G}}$, this holds because $\hat{U}$ acts on the vacuum state vector $|\mathbf{0}\rangle$ and any passive mode transformation maps the vacuum into itself. For the second setting, i.e., for $\varrho_{\mathrm{t}} \in \mathcal{C}_{\mathrm{LO}}$, this holds simply because there we assume that the total transformation itself is passive, i.e., in that case it holds also that $\mathbf{D} = \mathbb{1}$, so that $\mathbf{S} = \mathbf{O}$.

In both cases, coupling between different modes only takes place through the linear-optical network described by $\mathbf{O}$. A general circuit can couple all m modes with each other, meaning that the quadrature operators of each output mode are linear combinations of those of all m input modes. However, often, each mode is only coupled to at most $d \leq m$ other modes. In these situations, $\mathbf{O}$ is a sparse matrix with at most $4md$ non-zero elements. More precisely, the columns of $\mathbf{O}$ are given by $2m$ orthonormal vectors $(\mathbf{o}^{(k)})_{k\in[2m]}$ each having at most $2d$ non-zero entries. Furthermore, since the position and momentum of each mode is coupled to at most the $2d$ quadratures of the same d modes, each pair $\mathbf{o}^{(2j-1)}$ and $\mathbf{o}^{(2j)}$ shares the same *sparsity property*, i.e., $\mathbf{o}^{(2j-1)}$ and $\mathbf{o}^{(2j)}$ have at least $2(m-d)$ zero entries in common, for all $j \in [m]$.

Gaussian case. Using that in the Gaussian case $\mathbf{S} = \mathbf{O}\,\mathbf{D}$ and squaring equation (26) yields

$$\begin{aligned}\hat{\tilde{r}}^2 &= \hat{\mathbf{r}}^T\mathbf{O}\mathbf{D}^{-2}\mathbf{O}^{-1}\hat{\mathbf{r}} - 2\mathbf{x}^T\mathbf{O}\mathbf{D}^{-2}\mathbf{O}^{-1}\hat{\mathbf{r}} + \mathbf{x}^T\mathbf{O}\mathbf{D}^{-2}\mathbf{O}^{-1}\mathbf{x} &(2)\\ &= \mathrm{Tr}\left[\mathbf{O}\mathbf{D}^{-2}\mathbf{O}^T[\hat{\mathbf{r}}\hat{\mathbf{r}}^T - (2\hat{\mathbf{r}} - \mathbf{x})\mathbf{x}^T]\right], &(3)\end{aligned}$$

where $\mathbf{O}^{-1} = \mathbf{O}^T$ has been used and the trace is taken not over the Hilbert space but over the $2m \times 2m$ matrix with operators as entries. Combining supplementary equation (2) and equations (27) and (29) yields

$$F^{(0)} = 1 - \mathrm{Tr}\left[\mathbf{O}\mathbf{D}^{-2}\mathbf{O}^T[\langle\hat{\mathbf{r}}\hat{\mathbf{r}}^T\rangle_{\varrho_{\mathrm{P}}} - (2\langle\hat{\mathbf{r}}\rangle_{\varrho_{\mathrm{P}}} - \mathbf{x})\mathbf{x}^T]\right] + \frac{m}{2}. \tag{4}$$

Now we introduce the *first moment vector* $\boldsymbol{\gamma} \in \mathbb{R}^{2m}$ and the symmetric *second moment matrix* $\boldsymbol{\Gamma}^{(1)} \in \mathbb{R}^{2m\times 2m}$ of ϱ_{P}, with components

$$\gamma_l \coloneqq \langle\hat{r}_l\rangle_{\varrho_{\mathrm{P}}} \quad \text{and} \quad \Gamma^{(1)}_{l,l'} \coloneqq \left\langle\frac{\hat{r}_l\hat{r}_{l'} + \hat{r}_{l'}\hat{r}_l}{2}\right\rangle_{\varrho_{\mathrm{P}}}, \tag{5}$$

respectively. Since the matrix $\mathbf{O}\mathbf{D}^{-2}\mathbf{O}^T$ is symmetric, it holds that

$$\mathrm{Tr}\left[\mathbf{O}\mathbf{D}^{-2}\mathbf{O}^T[\langle\hat{\mathbf{r}}\hat{\mathbf{r}}^T\rangle_{\varrho_{\mathrm{P}}}\right] = \mathrm{Tr}\left[\mathbf{O}\mathbf{D}^{-2}\mathbf{O}^T[\langle\hat{\mathbf{r}}\hat{\mathbf{r}}^T\rangle^T_{\varrho_{\mathrm{P}}}\right], \tag{6}$$

so that we can rewrite supplementary equation (4) in terms of the observables which Arthur has access to as

$$F^{(0)} = 1 - \mathrm{Tr}\left[\mathbf{O}\mathbf{D}^{-2}\mathbf{O}^{-1}[\boldsymbol{\Gamma}^{(1)} - (2\boldsymbol{\gamma} - \mathbf{x})\mathbf{x}^T]\right] + \frac{m}{2}. \tag{7}$$

Supplementary Box 1 (Measurement scheme $\mathcal{M}_{\mathrm{G}}$).
First moment vector: For each $1 \leq l \leq 2m$, Arthur uses C_1 copies of ϱ_{P}, with C_1 given by supplementary equation (62a), to measure the observable $\hat{r}_l$, obtaining an estimate $\gamma_l^\star$ of the expectation value $\gamma_l = \langle \hat{r}_l \rangle_{\varrho_{\mathrm{P}}}$.
Second moment matrix: For each $1 \leq l \leq l' \leq 2m$ for which $(\mathbf{O}\mathbf{D}^{-2}\mathbf{O}^{-1})_{l,l'} = \sum_{k=1}^{2m} o_l^{(k)} D_{k,k}^{-2} o_{l'}^{(k)} \neq 0$, he uses C_2 copies of ϱ_{P}, with C_2 given by supplementary equation (62b), to measure the observable $\frac{1}{2}(\hat{r}_l\hat{r}_{l'} + \hat{r}_{l'}\hat{r}_l)$, obtaining an estimate $\Gamma_{l,l'}^{(1)\star}$ of the expectation values $\Gamma_{l,l'}^{(1)} = \Gamma_{l',l}^{(1)}$ in supplementary equation (5).
Classical post-processing: He obtains the estimate $F^{(0)\star}$ of $F^{(0)}$ by replacing in supplementary equation (7) the actual expectation values $\mathbf{\Gamma}^{(1)}$ and $\boldsymbol{\gamma}$ by the estimates $\Gamma^{(1)\star}$ and $\boldsymbol{\gamma}^\star$, respectively.

We will show later (see Supplementary Lemma 4 in Supplementary Note 3 and the discussion immediately after its proof) that the bound in supplementary equation (7) actually depends on at most $2m\kappa$ out of the $4m^2$ entries of $\mathbf{\Gamma}^{(1)}$, with $\kappa = 2\min\{d^2, m\}$, as defined in equation (7). Thus, only the $2m\kappa$ corresponding observables, and the $2m$ observables necessary for $\boldsymbol{\gamma}$, as indicated in Supplementary Box 1, need to be measured. All these observables can be measured by homodyne detection[6]. Furthermore, in Supplementary Note 4 we show that only $m+3$ different measurement settings are required. Finally, by classical post-processing, Arthur recombines his estimates according to the third step of Supplementary Box 1 and obtains the fidelity estimate $F^{(0)\star}$. This last step is also efficient in m.

Linear-optical case. For $\varrho_{\mathrm{t}} \in \mathcal{C}_{\mathrm{LO}}$ the unitary $\hat{U}$ is assumed to be passive. Hence, one has $\mathbf{x} = \mathbf{0}$ and $\mathbf{S} = \mathbf{O}$, and it follows that

$$\hat{\tilde{r}}^2 = \hat{r}^2 \,. \tag{8}$$

The components of $\tilde{r}$ are

$$\hat{\tilde{q}}_j = \mathbf{o}^{(2j-1)^T}\hat{\mathbf{r}} \quad \text{and} \quad \hat{\tilde{p}}_j = \mathbf{o}^{(2j)^T}\hat{\mathbf{r}}, \tag{9}$$

where $\mathbf{o}^{(k)}$ denotes the k-th column of $\mathbf{O}$. Defining

$$\mathbf{P}^{(j)} := \mathbf{o}^{(2j-1)}\mathbf{o}^{(2j-1)^T} + \mathbf{o}^{(2j)}\mathbf{o}^{(2j)^T} \tag{10}$$

as the projector onto the subspace spanned by the two vectors $\mathbf{o}^{(2j-1)}$ and $\mathbf{o}^{(2j)}$ and using supplementary equation (9) and equations (27) and (31), we obtain

$$F^{(n)} = \left\langle \left[n + 1 - \left(\hat{r}^2 - \frac{m}{2}\right)\right] \prod_{j=1}^{n} \left(\hat{\mathbf{r}}^T\mathbf{P}^{(j)}\hat{\mathbf{r}} - \frac{1}{2}\right) \right\rangle_{\varrho_{\mathrm{P}}} . \tag{11}$$

Next, we consider the $\binom{n}{j}$ subsets of $\{1, 2, \ldots, n\}$ of length j and define $\Omega_\mu^{(j)}$ as the μ-th of these subsets for some arbitrary ordering. With this, we expand the product inside supplementary equation (11) as

$$\prod_{j=1}^{n} \left(\hat{\mathbf{r}}^T\mathbf{P}^{(j)}\hat{\mathbf{r}} - \frac{1}{2}\right) = \sum_{j=0}^{n}(-1/2)^{n-j} \sum_{\mu=1}^{\binom{n}{j}} \bigotimes_{i\in\Omega_\mu^{(j)}} \hat{\mathbf{r}}^T\mathbf{P}^{(i)}\hat{\mathbf{r}}. \tag{12}$$

Using that a product of traces can be written as a trace over tensor products, supplementary equation (11) can be written as

$$F^{(n)} = \left\langle \left[n + 1 - \left(\hat{r}^2 - \frac{m}{2}\right)\right] \sum_{j=0}^{n}(-1/2)^{n-j} \sum_{\mu=1}^{\binom{n}{j}} \mathrm{Tr}\left[\left(\bigotimes_{i\in\Omega_\mu^{(j)}} \mathbf{P}^{(i)}\right)(\hat{\mathbf{r}}\hat{\mathbf{r}}^T)^{\otimes j}\right] \right\rangle_{\varrho_{\mathrm{P}}} , \tag{13}$$

where $\bigotimes_{i\in\Omega_\mu^{(0)}=\emptyset} \mathbf{P}^{(i)} := 1$ and the traces are again taken not over the Hilbert space but over tensors that have operators as components. For each $j \in [n+1]$, we introduce the $2j$*-th moment tensors* $\mathbf{\Gamma}^{(j)} \in (\mathbb{R}^{2m\times 2m})^{\otimes j}$ with components

$$\Gamma^{(j)}_{k_1,l_1,\ldots,k_j,l_j} := \left\langle \frac{\hat{r}_{k_1}\hat{r}_{l_1} + \hat{r}_{l_1}\hat{r}_{k_1}}{2} \cdots \frac{\hat{r}_{k_j}\hat{r}_{l_j} + \hat{r}_{l_j}\hat{r}_{k_j}}{2} \right\rangle_{\varrho_{\mathrm{P}}} \tag{14}$$

Supplementary Box 2 (Measurement scheme $\mathcal{M}_{\rm LO}$).

1) **2j-th and (2j+1)-th moment tensors**: For each $1 \leq j \leq n$, each $1 \leq \mu \leq \binom{n}{j}$, and each $1 \leq k_1, l_1, k_2, l_2, \ldots, k_j, l_j \leq 2m$, for which

$$\Big(\bigotimes_{i\in\Omega_\mu^{(j)}} \mathbf{P}^{(i)}\Big)_{k_1,l_1,k_2,l_2,\ldots,k_j,l_j} \neq 0, \tag{17}$$

Arthur uses $C_{\leq 2(n+1)}$ copies of $\varrho_{\rm p}$, with $C_{\leq 2(n+1)}$ given by supplementary equation (85), to measure the observable $(\hat{r}_{k_1}\hat{r}_{l_1} + \hat{r}_{l_1}\hat{r}_{k_1})/2 \cdots (\hat{r}_{k_j}\hat{r}_{l_j} + \hat{r}_{l_j}\hat{r}_{k_j})/2$, obtaining an estimate $\Gamma^{(j)\star}{}_{k_1,l_1,k_2,l_2,\ldots,k_j,l_j}$ of the $2j$-th moment $\Gamma^{(j)}_{k_1,l_1,k_2,l_2,\ldots,k_j,l_j}$. For each $1 \leq k_{j+1} \leq 2m$, he uses $C_{\leq 2(n+1)}$ copies of $\varrho_{\rm p}$ to measure the observable $((\hat{r}_{k_1}\hat{r}_{l_1} + \hat{r}_{l_1}\hat{r}_{k_1})/2) \cdots ((\hat{r}_{k_j}\hat{r}_{l_j} + \hat{r}_{l_j}\hat{r}_{k_j})/2)\hat{r}^2_{k_{j+1}}$, obtaining an estimate $\Gamma^{(j+1)\star}{}_{k_1,l_1,k_2,l_2,\ldots,k_j,l_j,k_{j+1},k_{j+1}}$ of the $2(j+1)$-th moment $\Gamma^{(j+1)}_{k_1,l_1,k_2,l_2,\ldots,k_j,l_j,k_{j+1},k_{j+1}}$.

2) **Classical post-processing**: He obtains the estimate $F^{(\mathbf{n})\star}$ of $F^{(n)}$ by replacing in supplementary equation (16) for all $1 \leq j \leq n+1$ the actual expectation values $\mathbf{\Gamma}^{(j)}$ by the estimates $\mathbf{\Gamma}^{(j)\star}$.

and define $\mathbf{\Gamma}^{(0)} := 1$. Clearly, these tensors are invariant under the partial transposition with respect to any j'-th pair of subindices $k_{j'}$ and $l_{j'}$,

$$\Gamma^{(j)}_{k_1,l_1,\ldots,k_{j'},l_{j'},\ldots,k_j,l_j} = \Gamma^{(j)}_{k_1,l_1,\ldots,l_{j'},k_{j'},\ldots,k_j,l_j}. \tag{15}$$

With the definition in supplementary equation (14) and the fact that each projector $\mathbf{P}^{(i)}$ is a symmetric matrix, supplementary equation (13) finally becomes

$$F^{(n)} = \sum_{j=0}^{n}(-1/2)^{n-j}\sum_{\mu=1}^{\binom{n}{j}}\left\{\left(n+1+\frac{m}{2}\right)\mathrm{Tr}\left[\Big(\bigotimes_{i\in\Omega_\mu^{(j)}}\mathbf{P}^{(i)}\Big)\mathbf{\Gamma}^{(j)}\right] - \mathrm{Tr}\left[\Big(\mathbb{1}\otimes\bigotimes_{i\in\Omega_\mu^{(j)}}\mathbf{P}^{(i)}\Big)\mathbf{\Gamma}^{(j+1)}\right]\right\}. \tag{16}$$

Note that this is an explicit expression for $F^{(n)}$ in terms of the correlators in supplementary equation (14) that Arthur can measure. Due to the sparsity of $\mathbf{O}$, each matrix $\mathbf{P}^{(i)}$ has at most $(2d)^2$ non-zero entries. Then, it follows (see Supplementary Lemma 7 in Supplementary Note 3 for details) that the measurement of $\mathrm{O}\left(m\left(4d^2+1\right)^n\right)$ observables, those listed in Supplementary Box 2, suffices for the estimation of the bound of supplementary equation (16). As in the Gaussian case, all these observables can be measured by homodyne detection[6]. Furthermore, in Supplementary Note 4 we show that at most $\binom{m}{n}2^{n+1} \leq (2m)^n/n!$ measurement settings are sufficient. Once again, by classical post-processing, Arthur recombines his estimates according to the third step of Supplementary Box 2 and obtains the fidelity estimate $F^{(n)\star}$. Provided that n is constant, this last step is also efficient in m.

Supplementary Note 2 - Quantum certification of locally post-selected target states.

In this note, we extend our results to locally post-selected $(m-a)$-mode target states $\varrho_{\mathcal{S}_{\rm t}}$ in $\mathcal{C}_{\rm PLO}$. The entire treatment of the class $\mathcal{C}_{\rm PLO}$ is similar to, and follows directly from, that already seen for the classes $\mathcal{C}_{\rm G}$ or $\mathcal{C}_{\rm LO}$. Therefore, instead of repeating all the details, we simply explain the specific differences.

The fidelity bound with post-selection. The first step is to derive the fidelity bound ${F_{\mathcal{S}}}^{(n)}$ given by equations (32) and (33). We proceed in a similar fashion to the Methods section in the main text. Due to equations (1) and (5), the facts that $\varrho_{\mathcal{S}_{\rm t}}$ and $\varrho_{\rm t}$

are pure, and the properties of the trace, it holds that

$$\begin{aligned} F_{\mathcal{S}} &= F(\varrho_{\mathcal{S}\mathrm{t}}, \varrho_{\mathcal{S}\mathrm{p}}) \\ &= \mathrm{Tr}_{\mathcal{S}}\left[\mathrm{Tr}_{\mathcal{A}}\left[\frac{\varrho_{\mathrm{t}}(\mathbb{1}_{\mathcal{S}} \otimes |\mathbf{n}_{\mathcal{A}}\rangle_{\mathcal{A}}\langle\mathbf{n}_{\mathcal{A}}|_{\mathcal{A}})}{\mathbb{P}(\mathbf{n}_{\mathcal{A}}|\varrho_{\mathrm{t}})}\right]\varrho_{\mathcal{S}\mathrm{p}}\right] \\ &= \frac{\mathrm{Tr}\left[\varrho_{\mathrm{t}}(\varrho_{\mathcal{S}\mathrm{p}} \otimes |\mathbf{n}_{\mathcal{A}}\rangle_{\mathcal{A}}\langle\mathbf{n}_{\mathcal{A}}|_{\mathcal{A}})\right]}{\mathbb{P}(\mathbf{n}_{\mathcal{A}}|\varrho_{\mathrm{t}})} \\ &= \frac{F\left(\varrho_{\mathrm{t}}, \varrho_{\mathcal{S}\mathrm{p}} \otimes |\mathbf{n}_{\mathcal{A}}\rangle_{\mathcal{A}}\langle\mathbf{n}_{\mathcal{A}}|_{\mathcal{A}}\right)}{\mathbb{P}(\mathbf{n}_{\mathcal{A}}|\varrho_{\mathrm{t}})}, \end{aligned} \tag{18}$$

where $\mathrm{Tr}_{\mathcal{S}}$ indicates partial trace over the Fock space of the $m-a$ modes in $\mathcal{S}$. Now, due to equation (27) with $\beta = n$, it holds that

$$F\left(\varrho_{\mathrm{t}}, \varrho_{\mathcal{S}\mathrm{p}} \otimes |\mathbf{n}_{\mathcal{A}}\rangle_{\mathcal{A}}\langle\mathbf{n}_{\mathcal{A}}|_{\mathcal{A}}\right) \geq \mathrm{Tr}\left[\hat{F}^{(n)}(\varrho_{\mathcal{S}\mathrm{p}} \otimes |\mathbf{n}_{\mathcal{A}}\rangle_{\mathcal{A}}\langle\mathbf{n}_{\mathcal{A}}|_{\mathcal{A}})\right] = \mathrm{Tr}_{\mathcal{S}}\left[\langle\mathbf{n}_{\mathcal{A}}|_{\mathcal{A}}\hat{F}^{(n)}|\mathbf{n}_{\mathcal{A}}\rangle_{\mathcal{A}}\,\varrho_{\mathcal{S}\mathrm{p}}\right], \tag{19}$$

with $\hat{F}^{(n)}$ the observable of equation (31). Using supplementary equations (18) and (19), we obtain the fidelity bound ${F_{\mathcal{S}}}^{(n)}$ of equations (32) and (33).

The derivation of the bound in supplementary equation (19) holds exactly the same if the m-mode target states $\varrho_{\mathrm{t}} \in \mathcal{C}_{\mathrm{LO}}$ are replaced by the more general target states $\varrho_{\mathrm{t}} = \hat{U}|\mathbf{n}\rangle\langle\mathbf{n}|\hat{U}^{\dagger}$, with $\hat{U}$ any Gaussian unitary and $|\mathbf{n}\rangle$ any Fock-basis state, and the ancilla Fock-basis state $|\mathbf{n}_{\mathcal{A}}\rangle_{\mathcal{A}}$ is replaced by any generic a-mode pure product state on the modes $\mathcal{A}$. What is not necessarily true for the more general post-selection scenario is that the resulting fidelity bound is tight for perfect experimental preparations. That is, there are post-selected target states for which the value of the resulting bound is too low to pass the certification test even if the preparation is perfect. Luckily, this does not happen for the experimentally more relevant case of linear-optical network target states post-selected with Fock-basis measurements, as we see in the following subsection.

Tightness of the fidelity bound for ideal experimental preparations with post-selection. Here, we show that the fidelity bound for linear-optical network target states post-selected with Fock-basis measurements is tight for ideal experimental preparations. That is, we show that, for $\varrho_{\mathcal{S}\mathrm{p}} = \varrho_{\mathcal{S}\mathrm{t}}$,

$$F_{\mathcal{S}}^{(n)} = F_{\mathcal{S}} = 1, \tag{20}$$

just as in the cases without post-selection.

We begin by expressing $F_{\mathcal{S}}^{(n)}$ as

$$\begin{aligned} F_{\mathcal{S}}^{(n)} &= \frac{\mathrm{Tr}\left[\hat{F}^{(n)}(\varrho_{\mathcal{S}\mathrm{p}} \otimes |\mathbf{n}_{\mathcal{A}}\rangle_{\mathcal{A}}\langle\mathbf{n}_{\mathcal{A}}|_{\mathcal{A}})\right]}{\mathbb{P}(\mathbf{n}_{\mathcal{A}}|\varrho_{\mathrm{t}})} \\ &= \frac{\mathrm{Tr}\left[\hat{F}^{(n)}\left(\frac{\langle\mathbf{n}_{\mathcal{A}}|_{\mathcal{A}}\varrho_{\mathrm{t}}|\mathbf{n}_{\mathcal{A}}\rangle_{\mathcal{A}}}{\mathbb{P}(\mathbf{n}_{\mathcal{A}}|\varrho_{\mathrm{t}})} \otimes |\mathbf{n}_{\mathcal{A}}\rangle_{\mathcal{A}}\langle\mathbf{n}_{\mathcal{A}}|_{\mathcal{A}}\right)\right]}{\mathbb{P}(\mathbf{n}_{\mathcal{A}}|\varrho_{\mathrm{t}})} \\ &= \frac{\frac{\langle\Psi_{\mathrm{t}}|(\mathbb{1}\otimes|\mathbf{n}_{\mathcal{A}}\rangle_{\mathcal{A}}\langle\mathbf{n}_{\mathcal{A}}|_{\mathcal{A}})}{\sqrt{\mathbb{P}(\mathbf{n}_{\mathcal{A}}|\varrho_{\mathrm{t}})}}\,\hat{U}\,(n+1-\hat{n})\prod_{j=1}^{n}\hat{n}_j\,\hat{U}^{\dagger}\,\frac{(\mathbb{1}\otimes|\mathbf{n}_{\mathcal{A}}\rangle_{\mathcal{A}}\langle\mathbf{n}_{\mathcal{A}}|_{\mathcal{A}})|\Psi_{\mathrm{t}}\rangle}{\sqrt{\mathbb{P}(\mathbf{n}_{\mathcal{A}}|\varrho_{\mathrm{t}})}}}{\mathbb{P}(\mathbf{n}_{\mathcal{A}}|\varrho_{\mathrm{t}})}. \end{aligned} \tag{21}$$

where, in the second equality, the assumption that $\varrho_{\mathcal{S}\mathrm{p}} = \varrho_{\mathcal{S}\mathrm{t}}$ and the definition of $\varrho_{\mathcal{S}\mathrm{t}}$ of equation (5) were used, and, in the third equality, equation (6) was used and the pure normalised target state vector

$$|\Psi_{\mathrm{t}}\rangle \coloneqq \hat{U}\,|\mathbf{1}_n\rangle \tag{22}$$

was introduced, i.e., such that $\varrho_{\mathrm{t}} = |\Psi_{\mathrm{t}}\rangle\langle\Psi_{\mathrm{t}}|$.

Now, due to equation (4), we know that $|\Psi_{\mathrm{t}}\rangle$ is an eigenstate of the total photon-number operator $\hat{n}$ with eigenvalue n. In addition, since $|\mathbf{n}_{\mathcal{A}}\rangle_{\mathcal{A}}$ is also an eigenstate of $\hat{n}$ and $\mathbb{P}(\mathbf{n}_{\mathcal{A}}|\varrho_{\mathrm{t}}) > 0$ by assumption, the normalised joint system-ancilla state after the projection,

$$|\psi_{\mathrm{t}}\rangle_{\mathcal{S}} \otimes |\mathbf{n}_{\mathcal{A}}\rangle_{\mathcal{A}} \coloneqq \frac{(\mathbb{1}_{\mathcal{S}} \otimes |\mathbf{n}_{\mathcal{A}}\rangle_{\mathcal{A}}\langle\mathbf{n}_{\mathcal{A}}|_{\mathcal{A}})\,|\Psi_{\mathrm{t}}\rangle}{\sqrt{\mathbb{P}(\mathbf{n}_{\mathcal{A}}|\varrho_{\mathrm{t}})}} = \frac{(\mathbb{1}_{\mathcal{S}} \otimes |\mathbf{n}_{\mathcal{A}}\rangle_{\mathcal{A}}\langle\mathbf{n}_{\mathcal{A}}|_{\mathcal{A}})\hat{U}\,|\mathbf{1}_n\rangle}{\sqrt{\langle\mathbf{1}_n|\,\hat{U}^{\dagger}(\mathbb{1} \otimes |\mathbf{n}_{\mathcal{A}}\rangle_{\mathcal{A}}\langle\mathbf{n}_{\mathcal{A}}|_{\mathcal{A}})\hat{U}\,|\mathbf{1}_n\rangle}}, \tag{23}$$

Supplementary Box 3 (Certification test $\mathcal{T}_{\mathrm{PLO}}$).
Settings adjustments: Idem as in $\mathcal{T}$ from Box 1.
State request: Arthur provides Merlin with the classical specification n, $\mathbf{S}$, a, and $|\mathbf{n}_{\mathcal{A}}\rangle_{\mathcal{A}}$ of the target state $\varrho_{\mathcal{S}_{\mathrm{t}}}$ and requests a sufficient number of copies of it.
Quantum measurements: He measures $\mathrm{O}\left(m(4d^2+1)^n\right)$ multi-body correlators, each one involving between 1 and $2n+1$ modes, specified by the measurement scheme $\mathcal{M}_{\mathrm{PLO}}$ (see "The measurement scheme with post-selection" below), which can be done with a single local heterodyne setting throughout.
Classical post-processing: By processing the measurement outcomes, he obtains a fidelity estimate $F_{\mathcal{S}}^{(n)\star}$ such that $F_{\mathcal{S}}^{(n)\star} \in \left[{F_{\mathcal{S}}}^{(n)} - \varepsilon, {F_{\mathcal{S}}}^{(n)} + \varepsilon\right]$ with probability at least $1-\alpha$, where ${F_{\mathcal{S}}}^{(n)}$ is the lower bound to $F_{\mathcal{S}}$ given by equations (32) and (33).
Accept-reject decision: If $F_{\mathcal{S}}^{(n)\star} < F_{\mathrm{T}} + \varepsilon$, he rejects. Otherwise, he accepts.

also turns out to be an eigenstate of $\hat{n}$ with eigenvalue n. Furthermore, since $\hat{U}^\dagger$ is passive, $\hat{U}^\dagger\,|\psi_{\mathrm{t}}\rangle_{\mathcal{S}} \otimes |\mathbf{n}_{\mathcal{A}}\rangle_{\mathcal{A}}$ is an eigenstate of $\hat{n}$ with eigenvalue n too and, therefore, eigenstate of $n+1-\hat{n}$ with eigenvalue 1. Thus, we can rewrite supplementary equation (21) as

$$F_{\mathcal{S}}^{(n)} = \frac{\langle\psi_{\mathrm{t}}|_{\mathcal{S}} \otimes \langle\mathbf{n}_{\mathcal{A}}|_{\mathcal{A}}\ \hat{U}\ \prod_{j=1}^{n}\hat{n}_j\ \hat{U}^\dagger\ |\psi_{\mathrm{t}}\rangle_{\mathcal{S}} \otimes |\mathbf{n}_{\mathcal{A}}\rangle_{\mathcal{A}}}{\mathbb{P}(\mathbf{n}_{\mathcal{A}}|\varrho_{\mathrm{t}})} = \frac{\langle\psi_{\mathrm{t}}|_{\mathcal{S}} \otimes \langle\mathbf{n}_{\mathcal{A}}|_{\mathcal{A}}\ \hat{U}\ |\mathbf{1}_n\rangle\langle\mathbf{1}_n|\ \hat{U}^\dagger\ |\psi_{\mathrm{t}}\rangle_{\mathcal{S}} \otimes |\mathbf{n}_{\mathcal{A}}\rangle_{\mathcal{A}}}{\mathbb{P}(\mathbf{n}_{\mathcal{A}}|\varrho_{\mathrm{t}})}, \tag{24}$$

where the second equality is due to the fact that, on the eigenvalue-n eigenspace of $\hat{n}$, the observable $\prod_{j=1}^{n}\hat{n}_j$ equals the projector $|\mathbf{1}_n\rangle\langle\mathbf{1}_n|$. Finally, using that $\mathbb{P}(\mathbf{n}_{\mathcal{A}}|\varrho_{\mathrm{t}}) = \mathrm{Tr}\left[\langle\mathbf{n}_{\mathcal{A}}|_{\mathcal{A}}\,\varrho_{\mathrm{t}}\,|\mathbf{n}_{\mathcal{A}}\rangle_{\mathcal{A}}\right]$, with ϱ_{t} defined by equation (4), and supplementary equation (23), one obtains that the right-hand side of supplementary equation (24) is equal to 1.

The certification test with post-selection. Next, in Supplementary Box 3, we present a test $\mathcal{T}_{\mathrm{PLO}}$ that works for post-selected target states in $\mathcal{C}_{\mathrm{PLO}}$ and which is a slightly modified version of the test $\mathcal{T}$ from Box 1. It is, of course, possible to unify both tests so as to account for all three classes of target states in one single test. We have, however, opted for splitting the tests into the two cases with and without post-selection to avoid an excessive notational overhead in Box 1.

The measurement scheme with post-selection. The measurement scheme to estimate ${F_{\mathcal{S}}}^{(n)}$ is essentially a replica of the scheme $\mathcal{M}_{\mathrm{LO}}$ to estimate $F^{(n)}$, already described in detail in the Supplementary Box 2. Thus, instead of repeating all the details, we simply outline the concrete differences between $\mathcal{M}_{\mathrm{LO}}$ and $\mathcal{M}_{\mathrm{PLO}}$. There are only three specific differences:

1. The moment tensors are now defined with respect to $\varrho_{\mathcal{S}_{\mathrm{p}}} \otimes |\mathbf{n}_{\mathcal{A}}\rangle_{\mathcal{A}}\langle\mathbf{n}_{\mathcal{A}}|_{\mathcal{A}}$ instead of ϱ_{p}. More precisely, we now need to estimate the tensors $\mathbf{\Gamma}_{\mathcal{S}}^{(j)} \in \left(\mathbb{R}^{2m\times 2m}\right)^{\otimes j}$, with elements
$$\Gamma_{\mathcal{S}\ k_1,l_1,\ldots,k_j,l_j}^{(j)} := \left\langle \frac{\hat{r}_{k_1}\hat{r}_{l_1}+\hat{r}_{l_1}\hat{r}_{k_1}}{2}\cdots\frac{\hat{r}_{k_j}\hat{r}_{l_j}+\hat{r}_{l_j}\hat{r}_{k_j}}{2}\right\rangle_{\varrho_{\mathcal{S}_{\mathrm{p}}}\otimes|\mathbf{n}_{\mathcal{A}}\rangle_{\mathcal{A}}\langle\mathbf{n}_{\mathcal{A}}|_{\mathcal{A}}} \tag{25}$$
$$= \left\langle \langle\mathbf{n}_{\mathcal{A}}|_{\mathcal{A}}\,\frac{\hat{r}_{k_1}\hat{r}_{l_1}+\hat{r}_{l_1}\hat{r}_{k_1}}{2}\cdots\frac{\hat{r}_{k_j}\hat{r}_{l_j}+\hat{r}_{l_j}\hat{r}_{k_j}}{2}\,|\mathbf{n}_{\mathcal{A}}\rangle_{\mathcal{A}}\right\rangle_{\varrho_{\mathcal{S}_{\mathrm{p}}}}. \tag{26}$$

2. ${F_{\mathcal{S}}}^{(n)}$ is obtained dividing the expression on the right-hand side of supplementary equation (16) by $\mathbb{P}(\mathbf{n}_{\mathcal{A}}|\varrho_{\mathrm{t}})$, and with $\mathbf{\Gamma}^{(j)}$ replaced by $\mathbf{\Gamma}_{\mathcal{S}}^{(j)}$.

3. The presence of the divisor $\mathbb{P}(\mathbf{n}_{\mathcal{A}}|\varrho_{\mathrm{t}})$ in ${F_{\mathcal{S}}}^{(n)}$ is the reason for the third difference. As discussed in Supplementary Lemma 11 in Supplementary Note 3, this divisor makes ${F_{\mathcal{S}}}^{(n)}$ more unstable than $F^{(n)}$ by a factor of $1/\mathbb{P}(\mathbf{n}_{\mathcal{A}}|\varrho_{\mathrm{t}})$. As a consequence, the number of copies of $\varrho_{\mathcal{S}_{\mathrm{p}}}$ required to estimate each relevant moment of ${F_{\mathcal{S}}}^{(n)}$ is $\frac{C_{\leq 2(n+1)}}{\mathbb{P}(\mathbf{n}_{\mathcal{A}}|\varrho_{\mathrm{t}})^2}$ instead of $C_{\leq 2(n+1)}$, as we discuss in Supplementary Lemma 12, also in Supplementary Note 3.

As is clear from supplementary equation (26), the estimation of $\mathbf{\Gamma}_{\mathcal{S}}^{(j)}$ requires only the measurement of multi-body correlators among the $(m-a)$ system modes in $\mathcal{S}$. This is due to the facts that, after the post-selection, the system is in a product state with respect to the bipartition $\mathcal{S}$ versus $\mathcal{A}$ (see supplementary equation (23)), and that the quadrature operators in supplementary equation (26) can also be correspondingly grouped into two factors, one containing exclusively operators of modes in $\mathcal{S}$ and the

other in $\mathcal{A}$. Furthermore, since $|\mathbf{n}_{\mathcal{A}}\rangle_{\mathcal{A}}$ is a product state known to Arthur, he can efficiently calculate the expectation vale of any product of quadrature operators of modes in $\mathcal{A}$ with respect to $|\mathbf{n}_{\mathcal{A}}\rangle_{\mathcal{A}}$. For instance, suppose that $k_1, l_1, k_2 \in \mathcal{A}$ and that $l_2, k_3, l_3 \ldots, k_j, l_j \notin \mathcal{A}$. Then, the corresponding $2j$-th moment decomposes as

$$\Gamma^{(j)}_{\mathcal{S}\ \ k_1,l_1,\ldots,k_j,l_j} = \langle \mathbf{n}_{\mathcal{A}}|_{\mathcal{A}} \frac{\hat{r}_{k_1}\hat{r}_{l_1} + \hat{r}_{l_1}\hat{r}_{k_1}}{2} \hat{r}_{k_2} |\mathbf{n}_{\mathcal{A}}\rangle_{\mathcal{A}} \left\langle \hat{r}_{l_2} \frac{\hat{r}_{k_3}\hat{r}_{l_3} + \hat{r}_{l_3}\hat{r}_{k_3}}{2} \cdots \frac{\hat{r}_{k_j}\hat{r}_{l_j} + \hat{r}_{l_j}\hat{r}_{k_j}}{2} \right\rangle_{\varrho_{\mathcal{S}_{\mathrm{p}}}}, \tag{27}$$

and only the measurement of the $(2j-3)$-th moment given by the second factor of supplementary equation (27) is required. As another example, consider the moments containing an odd number of quadrature operators of any $\mathcal{A}_j$-th mode. Since $|\mathbf{n}_{\mathcal{A}}\rangle_{\mathcal{A}}$ is a Fock-basis state, all these moments automatically vanish and need therefore not be measured at all.

Arthur can always efficiently obtain the $\mathbf{\Gamma}^{(j)}_{\mathcal{S}}$'s as a product of an (a priori known) expectation value with respect to $|\mathbf{n}_{\mathcal{A}}\rangle_{\mathcal{A}}$ of a multi-body product of quadrature operators of modes in $\mathcal{A}$ and a (measured) expectation value with respect to $\varrho_{\mathcal{S}_{\mathrm{p}}}$ of a multi-body product of quadrature operators of modes in $\mathcal{S}$, in a way analogous to the example of supplementary equation (27).

Formal statement of quantum certification of locally post-selected target states. Since the moments to be estimated, given in supplementary equation (26), are expectation values with respect to $\varrho_{\mathcal{S}_{\mathrm{p}}} \otimes |\mathbf{n}_{\mathcal{A}}\rangle_{\mathcal{A}} \langle \mathbf{n}_{\mathcal{A}}|_{\mathcal{A}}$, instead of ϱ_{p}, a simple way to extend Theorem 3 to target states in $\mathcal{C}_{\mathrm{PLO}}$ is by redefining the variance upper bounds σ_i. More precisely, taking σ_i as an upper bound on the variances of any product of i phase space quadratures now in the state $\varrho_{\mathcal{S}_{\mathrm{p}}}$, we introduce the quantities

$$\varsigma_i := \max_{j\in[a],\, k_1,k_2,\ldots k_j \in \mathcal{A}} \left\{ \langle \mathbf{n}_{\mathcal{A}}|_{\mathcal{A}}\, \hat{r}_{k_1}\hat{r}_{k_2}\ldots\hat{r}_{k_j}\, |\mathbf{n}_{\mathcal{A}}\rangle_{\mathcal{A}}\ \sigma_{i-j} \right\}, \tag{28}$$

for $i \in [2(n+1)]$. With them, we define $\varsigma_{\leq i} := \max_{k\leq i}\{\varsigma_k\}$ as the *maximal i-th generalised variance* of $\varrho_{\mathcal{S}_{\mathrm{p}}}$.

The parameters ς_i quantify the maximal variances of random variables defined by products of $i-j$ quadrature-measurement outcomes on $\varrho_{\mathcal{S}_{\mathrm{p}}}$ renormalised by the expectation value of products of j quadrature operators with respect to $|\mathbf{n}_{\mathcal{A}}\rangle_{\mathcal{A}}$, therefore automatically accounting for factorisations of the type of supplementary equation (27). They constitute non-tight upper bounds to the real variances. In particular experimental situations, tighter bounds can be found. Here, we are simply interested in taking advantage of the proof of Theorem 3 without introducing too much extra notational overhead, for which the definition of supplementary equation (28) is enough. Indeed, with these redefinitions, the following corollary follows straightforwardly from Theorem 3.

Supplementary Corollary 1 (Quantum certification of locally post-selected linear-optical network states). Under the same conditions and for the same ϱ_{t} as in Theorem 3, test $\mathcal{T}_{\mathrm{PLO}}$ from Supplementary Box 3 is a certification test for $\varrho_{\mathcal{S}_{\mathrm{t}}} \in \mathcal{C}_{\mathrm{PLO}}$ and requires at most

$$\mathrm{O}\left(\frac{\varsigma^2_{\leq 2(n+1)} m^4 (\lambda\, d^6\, n\, m)^n}{[\mathbb{P}(\mathbf{n}_{\mathcal{A}}|\varrho_{\mathrm{t}})\,\varepsilon]^2 \ln(1/(1-\alpha))} \right) \tag{29}$$

copies of a preparation $\varrho_{\mathcal{S}_{\mathrm{p}}}$ with maximal $2(n+1)$-th generalised variance $\varsigma_{\leq 2(n+1)}$, where $\lambda > 0$ is the same absolute constant as in Theorem 3.

Supplementary Corollary 1 is proven in Supplementary Note 3. Supplementary equation (29) corresponds to exactly the same expression as in equation (9) with the replacements $\sigma \to \varsigma$ and $\varepsilon \to \mathbb{P}(\mathbf{n}_{\mathcal{A}}|\varrho_{\mathrm{t}})\,\varepsilon$. The rescaling of ε with the factor $\mathbb{P}(\mathbf{n}_{\mathcal{A}}|\varrho_{\mathrm{t}})$ originates directly from the new expression for the fidelity given in supplementary equation (18), which renders the fidelity bound more unstable than $F^{(n)}$ by the factor of $1/\mathbb{P}(\mathbf{n}_{\mathcal{A}}|\varrho_{\mathrm{t}})$. In most interesting cases, the post-selection success probability $\mathbb{P}(\mathbf{n}_{\mathcal{A}}|\varrho_{\mathrm{t}})$ turns out to be exponentially small in a. Moreover, one can always come up with families of target states and post selection procedures for which $\mathbb{P}(\mathbf{n}_{\mathcal{A}}|\varrho_{\mathrm{t}})$ decreases arbitrarily fast in m. In such cases, the scaling in supplementary equation (29) is not efficient in m, inheriting the inefficiency of the state preparation by local measurements and post selection. However, the scaling is efficient in $1/\mathbb{P}(\boldsymbol{\phi}_{\mathcal{A}}|\varrho_{\mathrm{t}})$. That is, in every practical situation where state preparation via post selection is feasible, so is state certification. Interestingly, even for families of target states and post selection procedures for which $\mathbb{P}(\boldsymbol{\phi}_{\mathcal{A}}|\varrho_{\mathrm{t}})$ decays exponentially in a, the overall scaling (of the bound in supplementary equation (29)) with a is better than the scalings (of both the bounds in equation (9) and supplementary equation (29)) with n. Indeed, the bound in supplementary equation (29) grows, just like the bound in equation (9), faster than exponentially in n. Finally, the bound in supplementary equation (29) scales polynomially with all the other relevant parameters, including $1/\varepsilon$. Thus, arbitrary m-mode target states from the class $\mathcal{C}_{\mathrm{PLO}}$, with constant n, are certified by $\mathcal{T}_{\mathrm{PLO}}$ efficiently in m, $1/\mathbb{P}(\boldsymbol{\phi}_{\mathcal{A}}|\varrho_{\mathrm{t}})$, and all the other relevant parameters.

Formal statement of robust quantum certification of locally post-selected target states. Finally, we show that our certification test for the locally post-selected target states of the class $\mathcal{C}_{\mathrm{PLO}}$, $\mathcal{T}_{\mathrm{PLO}}$, is also robust. In a way analogous to equation (10),

we write $\varrho_{\mathcal{S}_{\mathrm{p}}}$ as

$$\varrho_{\mathcal{S}_{\mathrm{p}}} = F_{\mathcal{S}} \varrho_{\mathcal{S}_{\mathrm{t}}} + (1 - F_{\mathcal{S}}) \varrho_{\mathcal{S}_{\mathrm{t}}}^{\perp}, \tag{30}$$

where $\varrho_{\mathcal{S}_{\mathrm{t}}}^{\perp}$ is a normalised state such that $\mathrm{Tr}[\varrho_{\mathcal{S}_{\mathrm{t}}} \varrho_{\mathcal{S}_{\mathrm{t}}}^{\perp}] = 0$. Then, analogously to equation (12), we introduce the quantity

$$F_{\mathcal{S}_{\perp}}^{(n)} := \left\langle \hat{F}_{\mathcal{S}}^{(n)} \right\rangle_{\varrho_{\mathrm{t}}^{\perp}} , \tag{31}$$

where $\hat{F}_{\mathcal{S}}^{(n)}$ is the observable given in equation (33). With these definitions, the following corollary of Theorem 5 holds true.

Supplementary Corollary 2 (Robust quantum certification of locally post-selected states). Under the same conditions as in Corollary 1, test $\mathcal{T}_{\mathrm{PLO}}$ from Supplementary Box 3 is a robust certification test for $\varrho_{\mathcal{S}_{\mathrm{t}}} \in \mathcal{C}_{\mathrm{PLO}}$ with fidelity gap

$$\Delta_{\mathcal{S}} := \frac{2\varepsilon + F_{\mathcal{S}_{\perp}}^{(n)} (F_{\mathrm{T}} - 1)}{1 - F_{\mathcal{S}_{\perp}}^{(n)}}. \tag{32}$$

The proof is identical to the one of Theorem 5 presented in Supplementary Note 3 but with the replacements $F \to F_{\mathcal{S}}$, $F^{(n)} \to F_{\mathcal{S}}^{(n)}$, $F^{(n)\star} \to F_{\mathcal{S}}^{(n)\star}$, $F_{\perp}^{(n)} \to F_{\mathcal{S}_{\perp}}^{(n)}$ and $\Delta \to \Delta_{\mathcal{S}}$.

Supplementary Note 3 - Proofs of the theorems and corollaries.

Before going to the proofs, we devote two sections to establish necessary notation, review some known facts, and prove a general lemma.

Norms. Here, we introduce some helpful notation used in the proofs and review a few facts about norms on finite dimensional vector spaces. The *max norm* $\|\cdot\|_{\max}$ of a tensor is the largest of the absolute values of its entries. For a matrix $\mathbf{A}$, for example, $\|\mathbf{A}\|_{\max} := \max_{k,l} |A_{k,l}|$. For $p \in [1, \infty]$, we denote the *vector p-norm* of a vector $\mathbf{a}$ by $\|\mathbf{a}\|_p$ and the *Schatten p-norm* of a matrix $\mathbf{A}$ by $\|\mathbf{A}\|_p$, which is defined to be the vector p-norm of the vector of its singular values. For any matrix $\mathbf{A}$, we define $\mathrm{vec}(\mathbf{A})$ to be a vector containing all the entries of $\mathbf{A}$ (in some order). Then one can see that

$$\|\mathbf{A}\|_2 = \|\mathrm{vec}(\mathbf{A})\|_2 \tag{33}$$

and

$$\|\mathbf{A}\|_{\max} = \|\mathrm{vec}(\mathbf{A})\|_{\infty}. \tag{34}$$

For the vector and Schatten p-norm of vectors with N elements and $N \times N$ matrices, respectively, the following inequalities hold

$$\|\cdot\|_1 \leq \sqrt{N} \|\cdot\|_2 \leq N \|\cdot\|_{\infty}. \tag{35}$$

Because the Schatten ∞-norm is induced by the vector 2-norm, i.e.,

$$\|\mathbf{A}\|_{\infty} = \sup_{\mathbf{y}} \frac{\|\mathbf{A}\mathbf{y}\|_2}{\|\mathbf{y}\|_2}, \tag{36}$$

it follows that for any two vectors $\boldsymbol{\epsilon}$ and $\mathbf{x}$

$$\|\boldsymbol{\epsilon} \mathbf{x}^T\|_{\infty} \leq \|\boldsymbol{\epsilon}\|_2 \|\mathbf{x}\|_2. \tag{37}$$

Reliable estimation of expectation values from samples. We continue by proving a general large-deviation bound for estimates of expectation values from a finite number of measurements on independent copies, which we need for the proofs of Theorems 2 and 3.

Supplementary Lemma 3 (Reliable estimation of multiple expectation values from samples). Let $\sigma > 0$, ρ be a state, and let $\hat{A}_1, \dots, \hat{A}_N$ be observables with expectation values $A_i := \mathrm{Tr}\left[\rho \hat{A}_i\right]$ and variances bounded as $\mathrm{Tr}\left[\rho \hat{A}_i^2\right] - A_i^2 \leq \sigma^2$. For each $i \in [N]$ and χ, let $X_i^{(\chi)}$ be the random variable given by the measurement statistics of $\hat{A}_i$ on state ρ; such that, in particular, the

$\left(X_i^{(\chi)}\right)_{i,\chi}$ are independent random variables and the *finite sample average* over c measurements of $\hat{A}_i$ is the random variable

$$A_i^\star := \frac{1}{c}\sum_{\chi=1}^{c} X_i^{(\chi)}. \tag{38}$$

Then, the $\{A_i^\star\}_i$ are independent and, for every $\epsilon > 0$ and $\overline{\alpha} \in [1/2, 1)$, it holds that

$$\mathbb{P}\big[\forall i:\ |A_i^\star - A_i| \leq \varepsilon\big] \geq \overline{\alpha} \tag{39}$$

whenever

$$c \geq \frac{\sigma^2(N+1)}{\varepsilon^2 \ln(1/\overline{\alpha})}. \tag{40}$$

Proof. The sample averages $\{A_i^\star\}_i$ are independent by definition. By Chebyshev's inequality, it holds that

$$\forall\, i \in [c]: \quad \mathbb{P}\big[|A_i^\star - A_i| > \varepsilon\big] < \frac{\sigma^2}{c\varepsilon^2}. \tag{41}$$

Since the $\{A_i^\star\}_i$ are independent random variables, this yields

$$\mathbb{P}\big[\forall\, i:\ |A_i^\star - A_i| \leq \varepsilon\big] \geq \left(1 - \frac{\sigma^2}{c\varepsilon^2}\right)^N. \tag{42}$$

Finally,

$$\left(1 - \frac{\sigma^2}{c\varepsilon^2}\right)^N \geq \overline{\alpha} \tag{43}$$

is satisfied if

$$c \geq c_{\mathrm{opt}} := \left\lceil \frac{\sigma^2/\varepsilon^2}{1 - \overline{\alpha}^{1/N}} \right\rceil. \tag{44}$$

To finish the proof we upper bound

$$c_{\mathrm{opt}} = \left\lceil \frac{\sigma^2/\varepsilon^2}{1 - \mathrm{e}^{-\frac{\ln(1/\overline{\alpha})}{N}}} \right\rceil. \tag{45}$$

Using that (see Supplementary Note 6) for all $x \geq 0$

$$\frac{1}{1 - \mathrm{e}^{-1/x}} \leq x + \frac{1}{2+2x} + \frac{1}{2}, \tag{46}$$

it follows that

$$c_{\mathrm{opt}} \leq \frac{\sigma^2}{\varepsilon^2}\left(\frac{N}{\ln(1/\overline{\alpha})} + \frac{1}{2 + \frac{2N}{\ln(1/\overline{\alpha})}} + \frac{1}{2}\right). \tag{47}$$

To simplify the right-hand side of this inequality, we use that, since $\overline{\alpha} \geq \frac{1}{2} \geq \mathrm{e}^{-1}$, it holds that $\ln(1/\overline{\alpha}) \leq 1$ and, therefore, $2 + \frac{2N}{\ln(1/\overline{\alpha})} \geq 4$. So, using again that $\ln(1/\overline{\alpha}) \leq 1$, we finally arrive at

$$c_{\mathrm{opt}} \leq \frac{\sigma^2}{\varepsilon^2}\left(\frac{N}{\ln(1/\overline{\alpha})} + \frac{3}{4}\right) \leq \frac{\sigma^2(N+1)}{\varepsilon^2 \ln(1/\overline{\alpha})}. \tag{48}$$

□

Proof of Theorem 2 on certification of Gaussian states. We start by proving three auxiliary lemmas specific to the fidelity

bound $F^{(0)}$ for the Gaussian case.

The first lemma provides upper-bounds on the number of elements of $\mathbf{\Gamma}^{(1)}$ on which the fidelity bound $F^{(0)}$ depends.

Supplementary Lemma 4 (Sparsity of the Gaussian fidelity bound). $F^{(0)}$ depends on at most $2m\kappa$ elements of $\mathbf{\Gamma}^{(1)}$. We call these the *relevant elements* of $\mathbf{\Gamma}^{(1)}$.

Proof. Supplementary equation (7) can be written as

$$F^{(0)} = 1 + \frac{m}{2} + \mathbf{x}^T \mathbf{O}\mathbf{D}^{-2}\mathbf{O}^T(2\boldsymbol{\gamma} - \mathbf{x}) - \mathrm{Tr}\big[\mathbf{O}\mathbf{D}^{-2}\mathbf{O}^T\mathbf{\Gamma}\big]. \tag{49}$$

The last term can, in turn, be expressed as

$$\mathrm{Tr}\big[\mathbf{O}\mathbf{D}^{-2}\mathbf{O}^T\mathbf{\Gamma}^{(1)}\big] = \sum_{k=1}^{2m} D_{k,k}^{-2}(\mathbf{o}^{(k)})^T\mathbf{\Gamma}^{(1)}\mathbf{o}^{(k)} \tag{50}$$

$$= \mathrm{Tr}\Bigg[\sum_{j=1}^{m}\Big\{s_j^{-2}\mathbf{o}^{(2j-1)}(\mathbf{o}^{(2j-1)})^T + s_j^2\mathbf{o}^{(2j)}(\mathbf{o}^{(2j)})^T\Big\}\mathbf{\Gamma}^{(1)}\Bigg]. \tag{51}$$

Due to the sparsity of $\mathbf{O}$, as described in Supplementary Note 1, each matrix $s_j^{-2}\mathbf{o}^{(2j-1)}(\mathbf{o}^{(2j-1)})^T + s_j^2\mathbf{o}^{(2j)}(\mathbf{o}^{(2j)})^T$ has at most $4d^2$ non-zero elements. Hence, summing over j, we see that $F^{(0)}$ depends on at most $4m\min\{d^2, m\} = 2\kappa m$ elements of $\mathbf{\Gamma}^{(1)}$. $\square$

Note that the counting argument following supplementary equation (50) does not take into account the fact that $\mathbf{\Gamma}^{(1)}$ is symmetric. Taking this fact into account, we see that, from the $4d^2$ relevant elements of $\mathbf{\Gamma}^{(1)}$ that appear in each term of the trace in supplementary equation (50), only $d(2d+1)$ are independent. Thus, even though $2m\kappa$ entries of $\mathbf{\Gamma}^{(1)}$ contribute to $F^{(0)}$, only $m\min\{d(2d+1), 4m\} \leq 2m\kappa$ of them must actually be measured.

The second auxiliary lemma bounds the deviation of $F^{(0)\star}$ from $F^{(0)}$ in terms of the errors made in the estimation of the individual expectation values entering $F^{(0)}$.

Supplementary Lemma 5 (Stability of the Gaussian fidelity bound). Let $F^{(0)\star}$ be defined like $F^{(0)}$ in supplementary equation (7) but with $\boldsymbol{\gamma}$ and $\mathbf{\Gamma}^{(1)}$ replaced by $\boldsymbol{\gamma}^\star$ and $\mathbf{\Gamma}^{(1)\star}$ and let $\epsilon_{\max} := \|\boldsymbol{\gamma} - \boldsymbol{\gamma}^\star\|_{\max}$ and $\varepsilon_{\max}^{(1)} := \|\mathbf{\Gamma}^{(1)} - \mathbf{\Gamma}^{(1)\star}\|_{\max}$. Then

$$|F^{(0)} - F^{(0)\star}| \leq 2s_{\max}^2\left(\varepsilon_{\max}^{(1)}\sqrt{\kappa}m + \epsilon_{\max}\|\mathbf{x}\|_2\sqrt{2m}\right). \tag{52}$$

Proof. For convenience, we define the *error vector*

$$\boldsymbol{\epsilon} := \boldsymbol{\gamma} - \boldsymbol{\gamma}^\star \in \mathbb{R}^{2m} \tag{53}$$

and the *error matrix*

$$\mathcal{E}^{(1)} := \mathbf{\Gamma}^{(1)} - \mathbf{\Gamma}^{(1)\star}. \tag{54}$$

The fidelity estimation error can then be written as

$$F^{(0)} - F^{(0)\star} = \mathrm{Tr}\left[\mathbf{O}\mathbf{D}^{-2}\mathbf{O}^T(\mathcal{E}^{(1)} + 2\boldsymbol{\epsilon}\mathbf{x}^T)\right]. \tag{55}$$

Due to Hölder's inequality,

$$|F^{(0)} - F^{(0)\star}| \leq \|\mathbf{O}\mathbf{D}^{-2}\mathbf{O}^T\|_\infty \|\mathcal{E}^{(1)} + 2\boldsymbol{\epsilon}\mathbf{x}^T\|_1 \tag{56}$$

$$\leq \|\mathbf{D}^{-2}\|_\infty\left(\|\mathcal{E}^{(1)}\|_1 + 2\|\boldsymbol{\epsilon}\|_2\|\mathbf{x}\|_2\right), \tag{57}$$

where in the last step we have used the bound in supplementary equation (37). The second inequality in supplementary equation (35) implies that $\|\boldsymbol{\epsilon}\|_2 \leq \sqrt{2m}\|\boldsymbol{\epsilon}\|_\infty$. It remains to bound $\|\mathcal{E}\|_1$. To this end, we use the first inequality in supplementary equation (35) and supplementary equation (33) to arrive at

$$\|\mathcal{E}^{(1)}\|_1 \leq \sqrt{2m}\|\,\mathrm{vec}(\mathcal{E}^{(1)})\|_2. \tag{58}$$

According to Supplementary Lemma 4, $F^{(0)}$ depends on at most $2\kappa m$ entries of $\mathcal{E}^{(1)}$. Without loss of generality we can hence omit all other elements of $\mathcal{E}^{(1)}$ and thus take $\operatorname{vec}(\mathcal{E}^{(1)})$ as a vector with at most $2\kappa m$ elements. Using this fact and the second inequality in supplementary equation (35) we obtain

$$\|\mathcal{E}^{(1)}\|_1 \leq \sqrt{2m}\sqrt{2m\kappa}\|\operatorname{vec}(\mathcal{E}^{(1)})\|_\infty \tag{59}$$

$$= 2m\sqrt{\kappa}\|\mathcal{E}^{(1)}\|_{\max}, \tag{60}$$

where we have used supplementary equation (34) in the last equality. Finally, putting everything together and using that, by definition, $\|\mathbf{D}^{-2}\|_\infty = s_{\max}^2$, we arrive at the inequality in supplementary equation (52). □

The last auxiliary lemma shows that the estimate of the fidelity lower-bound for target states $\varrho_{\mathrm{t}} \in \mathcal{C}_{\mathrm{G}}$ obtained with the measurement scheme $\mathcal{M}_{\mathrm{G}}$ in Supplementary Box 1 is reliable. This lemma is potentially interesting in its own right in scenarios other than certification.

Supplementary Lemma 6 (Reliable estimation of the Gaussian fidelity bound). Let $\alpha \in (0, 1/2]$ and $\varepsilon > 0$. Let $F^{(0)\star}$ be defined like $F^{(0)}$ in supplementary equation (7) but with $\boldsymbol{\gamma}$ and $\boldsymbol{\Gamma}^{(1)}$ replaced by $\boldsymbol{\gamma}^\star$ and $\boldsymbol{\Gamma}^{(1)\star}$, where $\boldsymbol{\gamma}^\star$ and $\boldsymbol{\Gamma}^{(1)\star}$ are obtained as described by $\mathcal{M}_{\mathrm{G}}$ from

$$C = 2mC_1 + 2\kappa m C_2 \tag{61}$$

copies of ϱ_{p} , with C_1 and C_2 integers such that

$$C_1 \geq 2^6 \frac{\sigma_1^2(2m+1)\, m\, s_{\max}^4\, \|\mathbf{x}\|_2^2}{\varepsilon^2 \ln\left(\frac{1}{1-\alpha}\right)} \tag{62a}$$

and

$$C_2 \geq 2^5 \frac{\sigma_2^2(2\kappa m+1)\, m^2\, s_{\max}^4\, \kappa}{\varepsilon^2 \ln\left(\frac{1}{1-\alpha}\right)}. \tag{62b}$$

Then,

$$\mathbb{P}\left[|F^{(0)} - F^{(0)\star}| \leq \varepsilon\right] \geq 1 - \alpha. \tag{63}$$

Proof. Our proof strategy is to show that, with probability at least $1-\alpha$, the $2m$ elements of $\boldsymbol{\gamma}$ and the $2m\kappa$ relevant elements of $\boldsymbol{\Gamma}^{(1)}$ are estimated within additive errors bounded as

$$\epsilon_{\max} \leq \epsilon_{\max}^\star := \frac{\varepsilon}{4 s_{\max}^2 \|\mathbf{x}\|_2 \sqrt{2m}} \tag{64a}$$

and

$$\varepsilon_{\max}^{(1)} \leq \varepsilon_{\max}^{\star(1)} := \frac{\varepsilon}{4 s_{\max}^2 \sqrt{\kappa m}}. \tag{64b}$$

If the inequalities in supplementary equation (64) are fulfiled, then, due to Supplementary Lemma 5, it holds that $|F^{(0)} - F^{(0)\star}| \leq \varepsilon$.

Since all $2m$ estimates $\{\gamma_l^\star\}_l$ are sample averages over independent copies of ϱ_{p}, the measurement outcomes to obtain the $\{\gamma_l^\star\}_l$ are all independent random variables, for each l described by the same probability distribution. Furthermore, by assumption, the variances of these variables are all upper-bounded by σ_1. Analogously, the measurement outcomes to obtain all $2m\kappa$ relevant estimates $\{\Gamma_{l,l'}^{(1)\star}\}_{l,l'}$ are independent random variables with variances upper-bounded by σ_2 and described, for each l and l', by the same probability distribution. Hence, according to Supplementary Lemma 3, with the choice $\overline{\alpha} = \sqrt{1-\alpha}$, taking

$$C_1 \geq 2 \frac{\sigma_1^2(2m+1)}{{\epsilon_{\max}^\star}^2 \ln\left(\frac{1}{1-\alpha}\right)} \tag{65a}$$

and

$$C_2 \geq 2 \frac{\sigma_2^2(2\kappa m+1)}{{\varepsilon_{\max}^{\star(1)}}^2 \ln\left(\frac{1}{1-\alpha}\right)}, \tag{65b}$$

is sufficient for both

$$\mathbb{P}\left[\forall l: \ |\gamma_l^\star - \gamma_l| \leq \epsilon_{\max}^\star\right] \geq \sqrt{1-\alpha} \tag{66a}$$

and

$$\mathbb{P}\left[\forall\, \Gamma_{l,l'}^{(1)} \text{ relevant}: \ |\Gamma_{l,l'}^{(1)\star} - \Gamma_{l,l'}^{(1)}| \leq \varepsilon_{\max}^{\star(1)}\right] \geq \sqrt{1-\alpha}. \tag{66b}$$

Since the $\{\gamma_l^\star\}_l$ and the $\{\Gamma_{l,l'}^{(1)\star}\}_{l,l'}$ are independent random variables, supplementary equations (66) imply that

$$\mathbb{P}\left[\begin{array}{rcl} \forall l: \ |\gamma_l^\star - \gamma_l| & \leq & \epsilon_{\max}^\star \\ \text{and } \forall\, \Gamma_{l,l'}^{(1)} \text{ relevant}: \ |\Gamma_{l,l'}^{(1)\star} - \Gamma_{l,l'}^{(1)}| & \leq & \varepsilon_{\max}^{\star(1)} \end{array}\right] \geq 1-\alpha. \tag{67}$$

Finally, inserting the definitions of $\epsilon_{\max}^\star$ and $\varepsilon_{\max}^{\star(1)}$ in supplementary equations (64) into supplementary equations (65), we see that supplementary equations (62) are equivalent to supplementary equations (65). □

Now, we prove the theorem on quantum certification of Gaussian states.

Proof of Theorem 2. That the total number of copies of ϱ_{p} (see supplementary equation (61)) needed for the certification test is asymptotically upper-bounded by equation (8) can be verified by straightforward calculation using supplementary equations (62). It remains to show that (i) if $\varrho_{\mathrm{p}} = \varrho_{\mathrm{t}}$, then $\mathcal{T}$ accepts with probability at least $1-\alpha$, i.e.,

$$\mathbb{P}\left[F^{(0)\star} \geq F_T + \varepsilon\right] \geq 1-\alpha, \tag{68}$$

and (ii) if ϱ_{p} is such that $F < F_T$, then $\mathcal{T}$ rejects with probability at least $1-\alpha$, i.e.,

$$\mathbb{P}\left[F^{(0)\star} < F_{\mathrm{T}} + \varepsilon\right] \geq 1-\alpha. \tag{69}$$

To show (i), we first recall that, if $\varrho_{\mathrm{p}} = \varrho_{\mathrm{t}}$, $F^{(0)} = 1$. With this, supplementary equation (63) in Supplementary Lemma 6 implies that

$$\mathbb{P}\left[F^{(0)\star} \geq 1 - \varepsilon\right] \geq 1-\alpha. \tag{70}$$

Since, by assumption of the theorem, the total estimation error is such that $\varepsilon \leq \frac{1-F_{\mathrm{T}}}{2}$, it holds that $1-\varepsilon \geq F_T + \varepsilon$. Substituting the latter inequality into supplementary equation (70) yields supplementary equation (68).

To show (ii), we first note that, since $F^{(0)} \leq F$ for all ϱ_{p}, if $F < F_T$, then

$$F^{(0)} < F_{\mathrm{T}}. \tag{71}$$

On the other hand, supplementary equation (63) implies also that

$$\mathbb{P}\left[F^{(0)\star} \leq F^{(0)} + \varepsilon\right] \geq 1-\alpha. \tag{72}$$

Inserting supplementary equation (71) into supplementary equation (72) yields supplementary equation (69). □

Proof of Theorem 3 on certification of linear optical network states. We proceed analogously to the last section and present three auxiliary lemmas specific to the fidelity bound $F^{(n)}$ for the linear-optical case before proving Theorem 3.

To state the first lemma in a compact form we introduce the shorthand $\mathbf{\Gamma} := \left(\mathbf{\Gamma}^{(i)}\right)_{i=1,\ldots,n+1}$ for the collection of all the moment tensors $\mathbf{\Gamma}^{(i)}$. Analogously, the collection of all the estimates $\mathbf{\Gamma}^{(i)\star}$ of the moment tensors, defined in Supplementary Box 2, is denoted by $\mathbf{\Gamma}^\star := \left(\mathbf{\Gamma}^{(i)\star}\right)_{i=1,\ldots,n+1}$.

Supplementary Lemma 7 (Sparsity of the linear-optical fidelity bound). We write the fidelity bound $F^{(n)}$ given in supplementary equation (16) as

$$F^{(n)} = \sum_{j=0}^{n} (-1/2)^{n-j} f_j \left(\mathbf{\Gamma}^{(j)}, \mathbf{\Gamma}^{(j+1)}\right), \tag{73}$$

where, for each $j \in \{0, \dots, n\}$, f_j is the bilinear functional defined by

$$f_j \left(\mathbf{\Gamma}^{(j)}, \mathbf{\Gamma}^{(j+1)}\right) := \sum_{\mu=1}^{\binom{n}{j}} \left\{ \left(n + 1 + \frac{m}{2}\right) \operatorname{Tr}\left[\left(\bigotimes_{i \in \Omega_\mu^{(j)}} \mathbf{P}^{(i)} \right) \mathbf{\Gamma}^{(j)}\right] - \operatorname{Tr}\left[\left(\mathbb{1} \otimes \bigotimes_{i \in \Omega_\mu^{(j)}} \mathbf{P}^{(i)} \right) \mathbf{\Gamma}^{(j+1)}\right] \right\}. \tag{74}$$

For each j, the functional f_j depends on at most $\binom{n}{j}(2d)^{2j}$ elements of $\mathbf{\Gamma}^{(j)}$ and on at most $\binom{n}{j} 2m(2d)^{2j}$ elements of $\mathbf{\Gamma}^{(j+1)}$. We call these the *relevant elements for* f_j. Moreover, $F^{(n)}$ depends on at most

$$N_{\leq 2(n+1)} := (1 + 2m)(4d^2 + 1)^n \in \mathrm{O}\left(m \left(4d^2 + 1\right)^n\right) \tag{75}$$

elements of $\mathbf{\Gamma}$. We call these the *relevant elements* of $\mathbf{\Gamma}$.

The subindex "$\leq 2(n+1)$" in $N_{\leq 2(n+1)}$ makes reference to the fact that $2j$-th moments with $j \in [n+1]$ are taken into account.

Proof. Supplementary equations (73) and (74) can be checked by a straightforward calculation. We use again the sparsity of $\mathbf{O}$, i.e., the property that its columns $\mathbf{o}^{(2j-1)}$ and $\mathbf{o}^{(2j)}$ have at least $2(m-d)$ zero element in common. Hence, each of the symmetric matrices $\mathbf{P}^{(j)}$, defined in supplementary equation (10), has at most $(2d)^2$ non-zero elements. Consequently, the projectors $\bigotimes_{i \in \Omega_\mu^{(j)}} \mathbf{P}^{(i)}$ and $\mathbb{1} \otimes \bigotimes_{i \in \Omega_\mu^{(j)}} \mathbf{P}^{(i)}$ in supplementary equation (74) have at most $(2d)^{2j}$ and $2m(2d)^{2j}$ non-zero elements. This implies that the first trace inside the sum in supplementary equation (74) depends on at most $2m(2d)^{2j}$ elements of $\mathbf{\Gamma}^{(j+1)}$ and the second trace inside the sum on at most $(2d)^{2j}$ elements of $\mathbf{\Gamma}^{(j)}$. Hence, each f_j depends on at most $\binom{n}{j}(2d)^{2j}$ elements of $\mathbf{\Gamma}^{(j)}$ and on at most $\binom{n}{j}2m(2d)^{2j}$ elements of $\mathbf{\Gamma}^{(j+1)}$. This proves the statements on the sparsity of the functionals f_i. From this, it follows that $F^{(n)}$ depends on at most

$$\sum_{i=0}^{n} \left(\binom{n}{i} (2d)^{2i} + \binom{n}{i} 2m(2d)^{2i} \right) = (1 + 2m)(4d^2 + 1)^n \tag{76}$$

elements of $\mathbf{\Gamma}$ in total, where in the last step we have used the binomial theorem. □

It is important to mention that, as in Supplementary Lemma 4 for the Gaussian case, the symmetry in supplementary equation (15) of each $\mathbf{\Gamma}^{(j)}$ was not taken into account. Thus, even though the lemma gives the maximal total number of relevant elements that contribute to $F^{(n)}$, many of them are not independent and must therefore not be measured.

The second auxiliary lemma upper-bounds the deviation of $F^{(n)\star}$ from $F^{(n)}$ in terms of the errors made in the estimation of the expectation values entering $F^{(n)}$.

Supplementary Lemma 8 (Stability of the linear-optical fidelity bound). Let $F^{(n)\star}$ be defined like $F^{(n)}$ in supplementary equation (16) but with $\mathbf{\Gamma}$ replaced by $\mathbf{\Gamma}^\star$ and let $\varepsilon_{\max} := \|\mathbf{\Gamma} - \mathbf{\Gamma}^\star\|_{\max}$. Then

$$|F^{(n)} - F^{(n)\star}| \leq \varepsilon_{\max} \left(n + 1 + 5m/2\right) \left(1/2 + 2d\sqrt{2nm}\right)^n. \tag{77}$$

Proof. For convenience, we define, for each $j \in [n]$, the *error tensor*

$$\mathcal{E}^{(j)} := \Gamma^{(j)} - \Gamma^{(j)\star} \in \left(\mathbb{R}^{2m \times 2m}\right)^{\otimes j}. \tag{78}$$

Using supplementary equation (73) and the fact that f_j is linear, we write the fidelity estimation error as

$$F^{(n)} - F^{(n)\star} = \sum_{j=0}^{n} (-1/2)^{n-j} f_j \left(\mathcal{E}^{(j)}, \mathcal{E}^{(j+1)}\right), \tag{79}$$

Applying Hölder's inequality and using that the Schatten ∞-norm of a tensor product of projectors is bounded by 1 yields

$$|f_j\left(\mathcal{E}^{(j)},\mathcal{E}^{(j+1)}\right)| \le \binom{n}{j}\left(\|\tilde{\mathcal{E}}^{(j+1)}\|_1 + \left(n+1+\frac{m}{2}\right)\|\tilde{\mathcal{E}}^{(j)}\|_1\right), \tag{80}$$

where the matrix $\tilde{\mathcal{E}}^{(j)}$ is defined element-wise by $\tilde{\mathcal{E}}^{(j)}_{\mathbf{k}^{(j)},\mathbf{l}^{(j)}} := \mathcal{E}^{(j)}_{k_1,l_1,\ldots,k_j,l_j}$, where $\mathbf{k}^{(j)} := (k_1,\ldots,k_j)$ and $\mathbf{l}^{(j)} := (l_1,\ldots,l_j)$. Thanks to the first bound in supplementary equation (35) and supplementary equation (33), we arrive at

$$|f_j\left(\mathcal{E}^{(j)},\mathcal{E}^{(j+1)}\right)| \le \binom{n}{j}(2m)^{j/2}\left(\sqrt{2m}\|\mathrm{vec}(\tilde{\mathcal{E}}^{(j+1)})\|_2 + \left(n+1+\frac{m}{2}\right)\|\mathrm{vec}(\tilde{\mathcal{E}}^{(j)})\|_2\right). \tag{81}$$

According to Supplementary Lemma 7, f_j depends on at most $\binom{n}{j}2m(2d)^{2j}$ elements of $\tilde{\mathcal{E}}^{(j+1)}$ and on at most $\binom{n}{j}(2d)^{2j}$ of $\tilde{\mathcal{E}}^{(j)}$. Without loss of generality we can hence omit, in supplementary equation (81), all other elements in $\tilde{\mathcal{E}}^{(j)}$ and $\tilde{\mathcal{E}}^{(j+1)}$ and thus take $\mathrm{vec}(\tilde{\mathcal{E}}^{(j)})$ and $\mathrm{vec}(\tilde{\mathcal{E}}^{(j+1)})$ as vectors with at most $\binom{n}{j}(2d)^{2j}$ and $\binom{n}{j}2m(2d)^{2j}$ elements, respectively. Then the second bound in supplementary equation (35) yields

$$|f_j\left(\mathcal{E}^{(j)},\mathcal{E}^{(j+1)}\right)| \le \binom{n}{j}^{3/2}(2m)^{j/2}(2d)^j\left[2m\|\tilde{\mathcal{E}}^{(j+1)}\|_{\max} + \left(n+1+\frac{m}{2}\right)\|\tilde{\mathcal{E}}^{(j)}\|_{\max}\right]. \tag{82}$$

Next, from supplementary equation (79), it follows that

$$|F^{(n)} - F^{(n)\star}| \le \varepsilon_{\max}\left[\sum_{j=0}^{n}\binom{n}{j}^{3/2}(1/2)^{n-j}\left(\sqrt{2m}2d\right)^j \times (5m/2+n+1)\right]. \tag{83}$$

Finally, using $\binom{n}{j}^{1/2} \le n^{j/2}$ and the binomial formula, we obtain the inequality in supplementary equation (77). $\square$

The last auxiliary lemma shows that the estimate of the fidelity lower-bound for target states $\varrho_{\mathrm{t}} \in \mathcal{C}_{\mathrm{LO}}$ obtained with the measurement scheme $\mathcal{M}_{\mathrm{LO}}$ in Supplementary Box 2 is reliable. This lemma is potentially interesting in its own right in scenarios other than certification.

Supplementary Lemma 9 (Reliable estimation of the linear-optical fidelity bound). Let $\alpha \in (0,1/2]$ and $\varepsilon > 0$. Let $F^{(n)\star}$ be defined like $F^{(n)}$ in supplementary equation (16) but with $\mathbf{\Gamma}$ replaced by $\mathbf{\Gamma}^\star$, where $\mathbf{\Gamma}^\star$ is obtained as described by $\mathcal{M}_{\mathrm{LO}}$ from

$$C = N_{\le 2(n+1)}C_{\le 2(n+1)} \tag{84}$$

copies of ϱ_{p}, with $N_{\le 2(n+1)}$ an integer given by supplementary equation (75) and $C_{\le 2(n+1)}$ an integer given by

$$C_{\le 2(n+1)} \ge \frac{\sigma^2_{\le 2(n+1)}(N_{\le 2(n+1)}+1)}{\varepsilon^2\ln(1/(1-\alpha))}(n+1+5m/2)^2\left(1/2+2d\sqrt{2nm}\right)^{2n}. \tag{85}$$

Then,

$$\mathbb{P}\left[|F^{(n)} - F^{(n)\star}| \le \varepsilon\right] \ge 1-\alpha. \tag{86}$$

Proof. Our proof strategy is similar to that of Supplementary Lemma 6. That is, we show that, with probability at least $1-\alpha$, the $N_{\le 2(n+1)}$ relevant elements of $\mathbf{\Gamma}$ are estimated within additive errors bounded as

$$\varepsilon_{\max} \le \varepsilon^\star_{\max} := \frac{\varepsilon}{(n+1+5m/2)\left(1/2+2d\sqrt{2nm}\right)^n}. \tag{87}$$

If this inequality is fulfiled, then, due to Supplementary Lemma 8, it holds that $|F^{(n)} - F^{(n)\star}| \le \varepsilon$.

According to Supplementary Lemma 3, with the choice $\overline{\alpha} = 1-\alpha$, taking

$$C_{\le 2(n+1)} \ge \frac{\sigma^2_{\le 2(n+1)}(N_{\le 2(n+1)}+1)}{{\varepsilon^\star_{\max}}^2\ln(1/(1-\alpha))}. \tag{88}$$

is sufficient to get

$$\mathbb{P}\left[\forall\, \Gamma^{(i)}_{k_1,l_1,\ldots,k_i,l_i} \text{ relevant} : |\Gamma^{(i)*}_{k_1,l_1,\ldots,k_i,l_i} - \Gamma^{(i)}_{k_1,l_1,\ldots,k_i,l_i}| \leq \varepsilon^{\star}_{\max}\right] \geq 1-\alpha \tag{89}$$

Finally, inserting the definition in supplementary equation (87) of $\varepsilon^{\star}_{\max}$ into supplementary equation (88), we see that supplementary equation (86) is equivalent to supplementary equation (88). □

Now, we prove the theorem on quantum certification of linear-optical network states.

Proof of Theorem 3. The proof is analogous to the proof of Theorem 2, but with supplementary equation (84), equation (9), $F^{(n)}$, $F^{(n)\star}$, Supplementary Lemma 9, and supplementary equation (86) playing respectively the roles of supplementary equation (61), equation (8) , $F^{(0)}$, $F^{(0)\star}$, Supplementary Lemma 6 and supplementary equation (63). □

Proof of Supplementary Corollary 1 on certification of post-selected target states. The proof relies on three auxiliary lemmas, equivalent to Supplementary Lemmas 7, 8, and 9. The proofs of the following lemmas are analogous to, and follow immediately from, those of the latter.

Supplementary Lemma 10 (Sparsity of the locally post-selected linear-optical fidelity bound). The fidelity bound ${F_{\mathcal{S}}}^{(n)}$, defined by the same expression as $F^{(n)}$ in supplementary equation (16) but divided by $\mathbb{P}(\mathbf{n}_{\mathcal{A}}|\varrho_{\mathrm{t}})$ and with $\mathbf{\Gamma}$ replaced by $\mathbf{\Gamma}_{\mathcal{S}}$, can be written as

$${F_{\mathcal{S}}}^{(n)} = \frac{1}{\mathbb{P}(\mathbf{n}_{\mathcal{A}}|\varrho_{\mathrm{t}})}\left[1-\sum_{j=0}^{n}(-1/2)^{n-j} f_j\left(\mathbf{\Gamma}_{\mathcal{S}}^{(j)}, \mathbf{\Gamma}_{\mathcal{S}}^{(j+1)}\right)\right], \tag{90}$$

where, for each $j \in \{0,\ldots,n\}$, f_j is the same linear functional as in Supplementary Lemma 7, defined by supplementary equation (74). Moreover, ${F_{\mathcal{S}}}^{(n)}$ depends on at most $N_{\leq 2(n+1)}$ elements of $\mathbf{\Gamma}_{\mathcal{S}}$, with $N_{\leq 2(n+1)}$ the same as in Supplementary Lemma 7 and given by supplementary equation (75).

Proof. The proof of the lemma is analogous to that of Supplementary Lemma 7. □

Supplementary Lemma 11 (Stability of the locally post-selected linear-optical fidelity bound). Let $F_{\mathcal{S}}^{(n)\star}$ be defined by the same expression as $F^{(n)}$ in supplementary equation (16) but divided by $\mathbb{P}(\mathbf{n}_{\mathcal{A}}|\varrho_{\mathrm{t}})$ and with $\mathbf{\Gamma}$ replaced by $\mathbf{\Gamma}^{\star}_{\mathcal{S}}$, and let $\varepsilon_{\max} := \|\mathbf{\Gamma}_{\mathcal{S}} - \mathbf{\Gamma}^{\star}_{\mathcal{S}}\|_{\max}$. Then

$$|{F_{\mathcal{S}}}^{(n)} - F_{\mathcal{S}}^{(n)\star}| \leq \frac{\varepsilon_{\max}}{\mathbb{P}(\mathbf{n}_{\mathcal{A}}|\varrho_{\mathrm{t}})}\,(n+1+5m/2)\left(1/2+2d\sqrt{2nm}\right)^{n}. \tag{91}$$

Proof. The proof of the lemma is similar to that of Supplementary Lemma 8, with the differences already explained in "The measurement scheme with post-selection" in Supplementary Note 2. □

Supplementary Lemma 12 (Reliable estimation of the locally post-selected linear-optical fidelity bound). Let $\alpha \in (0,1/2]$ and $\varepsilon > 0$. Let $F_{\mathcal{S}}^{(n)\star}$ be defined like $F^{(n)}$ in supplementary equation (16) but with $\mathbf{\Gamma}$ replaced by $\mathbf{\Gamma}^{\star}_{\mathcal{S}}$, where $\mathbf{\Gamma}^{\star}_{\mathcal{S}}$ is obtained as described in "The measurement scheme with post-selection" in Supplementary Note 2 from

$$C = N_{\leq 2(n+1)} C_{\leq 2(n+1)} \tag{92}$$

copies of $\varrho_{\mathcal{S}_{\mathrm{p}}}$, with $N_{\leq 2(n+1)}$ an integer given by supplementary equation (75) and $C_{\leq 2(n+1)}$ an integer given by

$$C_{\leq 2(n+1)} \geq \frac{\varsigma^2_{\leq 2(n+1)}(N_{\leq 2(n+1)}+1)}{\left[\mathbb{P}(\mathbf{n}_{\mathcal{A}}|\varrho_{\mathrm{t}})\,\varepsilon\right]^2 \ln(1/(1-\alpha))}\,(n+1+5m/2)^2\left(1/2+2d\sqrt{2nm}\right)^{2n}. \tag{93}$$

Then,

$$\mathbb{P}\left[|{F_{\mathcal{S}}}^{(n)} - F_{\mathcal{S}}^{(n)\star}| \leq \varepsilon\right] \geq 1-\alpha. \tag{94}$$

Proof. The proof of the lemma is analogous to that of Supplementary Lemma 9. □

Proof of Supplementary Corollary 1. The proof is analogous to the proof of Theorem 3 but with Supplementary Lemmas 10, 11, and 12 playing respectively the roles of Supplementary Lemmas 7, 8, and 9. □

Proof of Supplementary Theorem 5 on robust quantum certification of post-selected target states. Crucial for the proof of this theorem is the expansion in equation (10) of ϱ_{P} in terms of ϱ_{t} and $\varrho_{\mathrm{t}}^{\perp}$, which allows for the definition of the parameter $F_{\perp}^{(n)}$ in equation (12).

Proof of Supplementary Theorem 5. Theorems 2 and 3 imply that ϱ_{P} is rejected with probability at least $1-\alpha$ whenever $F < F_{\mathrm{T}}$. Thus, it remains to show that if ϱ_{P} is such that $F \geq F_{\mathrm{T}} + \Delta$, with Δ given by equation (13), then ϱ_{P} is accepted with probability at least $1 - \alpha$, i.e., that

$$\mathbb{P}\left[F^{(n)\star} \geq F_{\mathrm{T}} + \varepsilon\right] \geq 1 - \alpha. \tag{95}$$

In turn, from supplementary equation (86), we know that supplementary equation (95) is satisfied if

$$F^{(n)} \geq F_{\mathrm{T}} + 2\varepsilon. \tag{96}$$

So, we let $F \geq F_{\mathrm{T}} + \Delta$, with Δ given by equation (13), and prove supplementary equation (96).

Using equations (6), (10), and (12), we write $F^{(n)}$ as

$$F^{(n)} = F + (1 - F)F_{\perp}^{(n)} = F\left(1 - F_{\perp}^{(n)}\right) + F_{\perp}^{(n)} \geq (F_{\mathrm{T}} + \Delta)\left(1 - F_{\perp}^{(n)}\right) + F_{\perp}^{(n)}, \tag{97}$$

where the inequality holds because $F \geq F_{\mathrm{T}} + \Delta$ by assumption and because $1 - F_{\perp}^{(n)} \geq 1$, since $F_{\perp}^{(n)} \leq 0$. Using equation (13) we see that

$$(F_{\mathrm{T}} + \Delta)\left(1 - F_{\perp}^{(n)}\right) + F_{\perp}^{(n)} = F_{\mathrm{T}} + 2\varepsilon. \tag{98}$$

Substituting supplementary equation (98) into the right-hand side of the inequality in supplementary equation (97) finishes the proof. □

Supplementary Note 4 - Number of measurement settings.

In this section, we upper-bound the number of local measurement settings required for the estimation of our fidelity lower bounds. We do this explicitly only for the Gaussian and linear-optical network target states, the cases of the post-selected target states following immediately from them.

Gaussian case. Here, we show that the $2md$ single-quadrature and the $m\kappa$ two-quadrature observables listed in Supplementary Box 1, required for the measurement scheme $\mathcal{M}_{\mathrm{G}}$, can all be measured using $m+3$ different experimental arrangements using homodyne detection. We do this by explicitly describing a homodyne measurement strategy that features such a scaling. We note that, alternatively, all the observables required for $\mathcal{M}_{\mathrm{G}}$ can also be measured with a single experimental arrangement throughout using heterodyne detection. The measurement procedure for heterodyning is nevertheless the same as in the linear-optical case below. Therefore, here we only describe the procedure with homodyning.

Any single-mode phase-space quadrature operator can be directly measured with homodyne detection[6,11–13]. The two-body observables $\hat{q}_j\hat{q}_k$, $\hat{q}_j\hat{p}_k$, and $\hat{p}_j\hat{p}_k$, for $j \neq k$, can be measured by simultaneously homodyning modes j and k independently. For all possible pairs of modes, this consumes $m+2$ different homodyne settings: A single setting $(\hat{q}_1, \hat{q}_2, \ldots, \hat{q}_m)$ for all the second moments of the form $\langle\hat{q}_j\hat{q}_k\rangle_{\varrho_{\mathrm{P}}}$; another single setting $(\hat{p}_1, \hat{p}_2, \ldots, \hat{p}_m)$ for those of the form $\langle\hat{p}_j\hat{p}_k\rangle_{\varrho_{\mathrm{P}}}$; and the m settings $(\hat{p}_1, \hat{q}_2, \ldots, \hat{q}_m)$, $(\hat{q}_1, \hat{p}_2, \hat{q}_3, \ldots, \hat{q}_m)$, …, and $(\hat{q}_1, \ldots, \hat{q}_{m-1}, \hat{p}_m)$ for those of the form $\langle\hat{q}_j\hat{p}_k\rangle_{\varrho_{\mathrm{P}}}$ and $\langle\hat{p}_j\hat{q}_k\rangle_{\varrho_{\mathrm{P}}}$ with $j \neq k$. In addition, all the single-body observables $\hat{q}_j$, $\hat{p}_j$, $\hat{q}_j^2$, and $\hat{p}_j^2$, are measured also with these same settings. With this, we have accounted, so far, for all the first moments γ_l and all the second moments $\Gamma_{l,l'}^{(1)}$ with $(l, l') \neq (2j-1, 2j)$ for all $j \in [m]$.

The remaining second moments, $\Gamma_{2j-1,2j}^{(1)}$ with $j \in [m]$, correspond to the single-mode observables $(\hat{q}_j\hat{p}_j + \hat{p}_j\hat{q}_j)/2$. To measure these, Arthur can homodyne each mode j independently in the rotated quadrature $(\hat{q}_j + \hat{p}_j)/\sqrt{2}$. This requires a single setting: $\left[(\hat{q}_1 + \hat{p}_1)/\sqrt{2}, (\hat{q}_2 + \hat{p}_2)/\sqrt{2}, \ldots, (\hat{q}_1 + \hat{p}_1)/\sqrt{2}\right]$. In this setting, he can estimate all the moments of the form $\langle(\hat{q}_j + \hat{p}_j)^2/2\rangle_{\varrho_{\mathrm{P}}}$. The latter estimates, upon subtraction of $\langle\hat{q}_j^2\rangle_{\varrho_{\mathrm{P}}}/2$ and $\langle\hat{p}_j^2\rangle_{\varrho_{\mathrm{P}}}/2$, whose settings have already been accounted for, finally make it possible to indirectly estimate $\langle(\hat{q}_j\hat{p}_j + \hat{p}_j\hat{q}_j)/2\rangle_{\varrho_{\mathrm{P}}}$, using the equation

$$\frac{1}{2}(\hat{q}_j\hat{p}_j + \hat{p}_j\hat{q}_j) = \left(\frac{\hat{q}_j + \hat{p}_j}{\sqrt{2}}\right)^2 - \frac{\hat{q}_j^2}{2} - \frac{\hat{p}_j^2}{2}. \tag{99}$$

The last setting, plus the $m+2$ ones already accounted for in the previous paragraph, yields a total of $m+3$ different homodyne

settings, as promised.

Finally, a comment on the error estimation is in order. In any measurement strategy where moments are estimated indirectly, their errors must be obtained from those of the directly measured quantities via error propagation. For instance, in the strategy just described, the error of each $\Gamma^{(1)}_{2j-1,2j}$ needs to be calculated from those of $\langle(\hat{q}_j+\hat{p}_j)^2/2\rangle_{\varrho_{\rm P}}$, $\langle\hat{q}_j^2\rangle_{\varrho_{\rm P}}$, and $\langle\hat{p}_j^2\rangle_{\varrho_{\rm P}}$. This leads, for each indirectly estimated moment, to an increase in the number of copies of $\varrho_{\rm P}$ required to attain a given error. Nevertheless, this usually has no impact on the leading terms of the total resource scaling of the protocol. For example, in the described strategy, the global scaling given in equation (8) remains unaltered.

Linear-optical case. Here, we show that the $N_{\leq 2(n+1)} \in \mathrm{O}\left(m\left(4d^2+1\right)^n\right)$ observables listed in Supplementary Box 2, required for the measurement scheme $\mathcal{M}_{\rm LO}$, can all be measured using a single experimental arrangement throughout using heterodyne detection.

Before that, however, we note that the majority of the these observables can be efficiently measured with homodyne detection in a similar fashion to that described in the Gaussian case above. Namely, the scheme $\mathcal{M}_{\rm LO}$ requires the measurement of products of an even number between 2 and $2(n+1)$ of quadrature operators. For arbitrary n, all products of $2n$ quadrature operators for which each relevant j-th mode contributes exclusively either with powers of a single quadrature ($\hat{q}_j$ or $\hat{p}_j$) or with the quadratic polynomial $(\hat{q}_j\hat{p}_j+\hat{p}_j\hat{q}_j)/2$ can be measured with essentially the same homodyne procedure as the one described in the Gaussian case above. One can show that this can be done with at most $\mathrm{O}(\binom{m}{n})$ different homodyne settings. This suffices to efficiently estimate the majority of the moments $\mathbf{\Gamma}^{(n)}$. However, the indirect estimation of higher-order moments, such as those involving powers of $\hat{q}_j\hat{p}_j+\hat{p}_j\hat{q}_j$, can be more involved. For example, using operator identities of the type of supplementary equation (99) and the canonical commutation relations, one can see that

$$(\hat{q}_j\hat{p}_j+\hat{p}_j\hat{q}_j)^2 = \frac{1}{3}\left[(\hat{q}_j+\hat{p}_j)^4+(\hat{q}_j-\hat{p}_j)^4\right]-\frac{2}{3}\left(\hat{q}_j^4+\hat{p}_j^4\right)+1, \tag{100a}$$

$$\hat{q}_j^2\hat{p}_j^2+\hat{p}_j^2\hat{q}_j^2 = \frac{1}{6}\left[(\hat{q}_j+\hat{p}_j)^4+(\hat{q}_j-\hat{p}_j)^4\right]-\frac{1}{3}\left(\hat{q}_j^4+\hat{p}_j^4\right)-1. \tag{100b}$$

This implies that the quartic polynomials in supplementary equations (100) can be indirectly estimated through homodyne measurements in the four local settings $\hat{q}_j$, $\hat{p}_j$, $\frac{\hat{q}_j+\hat{p}_j}{\sqrt{2}}$, and $\frac{\hat{q}_j-\hat{p}_j}{\sqrt{2}}$, instead of just three as in the quadratic case. In general, the number of homodyne settings required for the indirect estimation of polynomials depending on both quadratures of a same mode grows with its order[14]. In return for that, the moments involving those polynomials require homodyne measurements on fewer modes. However, it is still useful to have another alternative. Heterodyning provides such an alternative.

Independent heterodyne detection on each mode requires a single experimental setting throughout. It implements –ideally– the generalised measurement $\left\{\frac{1}{\pi^{m/2}}\,|\boldsymbol{\alpha}\rangle\langle\boldsymbol{\alpha}|\right\}_{\boldsymbol{\alpha}\in\mathbb{C}^m}$, where $|\boldsymbol{\alpha}\rangle$ is the m-mode coherent state of amplitude $\boldsymbol{\alpha} := (\alpha_1,\alpha_2,\ldots,\alpha_m)$[6,11–13]. That is, for arbitrary $\varrho_{\rm P}$, heterodyne detection outputs the amplitude $\boldsymbol{\alpha}\in\mathbb{C}^m$ with a probability

$$\mathbb{P}(\boldsymbol{\alpha}) = \mathrm{Tr}\left[\frac{1}{\pi^m}\,|\boldsymbol{\alpha}\rangle\langle\boldsymbol{\alpha}|\;\varrho_{\rm P}\;|\boldsymbol{\alpha}\rangle\langle\boldsymbol{\alpha}|\right] =: Q_{\varrho_{\rm P}}(\boldsymbol{\alpha},\boldsymbol{\alpha}^*), \tag{101}$$

where the Husimi Q function $Q_{\varrho_{\rm P}}$ of $\varrho_{\rm P}$ has been introduced. The Q function is such that, for any $\varrho_{\rm P}$, it holds that $0\leq Q_{\varrho_{\rm P}}(\boldsymbol{\alpha},\boldsymbol{\alpha}^*)\leq\frac{1}{\pi^m}$ for all $\boldsymbol{\alpha}\in\mathbb{C}^m$, so that it yields a well-defined classical phase-space probability distribution. It provides an experimentally convenient tool for the evaluation of expectation values of anti-normal ordered observables, i.e., observables expressed with all annihilation operators to the left of all creation operators[11–13].

Let us represent the anti-normal ordered expression of an operator by attaching two dots around it. For instance, we write

$$\hat{q}_j\hat{p}_j+\hat{p}_j\hat{q}_j = \frac{i}{2}\left[\hat{a}_j^2-(\hat{a}_j^\dagger)^2\right] =: \cdot\hat{q}_j\hat{p}_j+\hat{p}_j\hat{q}_j\cdot, \tag{102}$$

$$\hat{q}_i^2\hat{p}_i+\hat{p}_i\hat{q}_i^2 = \frac{i}{2}\left[\hat{a}_j-\hat{a}_j^\dagger+\hat{a}_j^3-(\hat{a}_j^\dagger)^3+\hat{a}_j^2\hat{a}_j^\dagger-\hat{a}_j(\hat{a}_j^\dagger)^2\right] =: \cdot\hat{q}_i^2\hat{p}_i+\hat{p}_i\hat{q}_i^2\cdot, \tag{103}$$

where the definitions $\hat{q}_j := \frac{\hat{a}_j+\hat{a}_j^\dagger}{\sqrt{2}}$ and $\hat{p}_j := i\frac{\hat{a}_j^\dagger-\hat{a}_j}{\sqrt{2}}$ and the commutation relations $[\hat{a}_j,\hat{a}_j^\dagger]=\mathbb{1}$ have been used. Moreover, let us denote by $\cdot\Gamma^{(j)}_{k_1,l_1,\ldots,k_j,l_j}\cdot(\hat{\boldsymbol{a}},\hat{\boldsymbol{a}}^\dagger)$, with $\hat{\boldsymbol{a}} := (\hat{a}_1,\hat{a}_2,\ldots,\hat{a}_m)$ and $\hat{\boldsymbol{a}}^\dagger := (\hat{a}_1^\dagger,\hat{a}_2^\dagger,\ldots,\hat{a}_m^\dagger)$, the anti-normal ordered expression of the observable in the expectation value of supplementary equation (14),

$$\cdot\,\Gamma^{(j)}_{k_1,l_1,\ldots,k_j,l_j}\cdot(\hat{\boldsymbol{a}},\hat{\boldsymbol{a}}^\dagger) := \cdot\left(\frac{\hat{r}_{k_1}\hat{r}_{l_1}+\hat{r}_{l_1}\hat{r}_{k_1}}{2}\ldots\frac{\hat{r}_{k_j}\hat{r}_{l_j}+\hat{r}_{l_j}\hat{r}_{k_j}}{2}\right)\cdot\,. \tag{104}$$

We emphasise that $\cdot\Gamma^{(j)}_{k_1,l_1,\ldots,k_j,l_j}\cdot(\hat{\boldsymbol{a}},\hat{\boldsymbol{a}}^\dagger)$ and $\frac{\hat{r}_{k_1}\hat{r}_{l_1}+\hat{r}_{l_1}\hat{r}_{k_1}}{2}\ldots\frac{\hat{r}_{k_j}\hat{r}_{l_j}+\hat{r}_{l_j}\hat{r}_{k_j}}{2}$ are the same observable. The former is just an

alternative expression (the anti-normal ordered one, in terms of annihilation and creation operators) of the latter (in terms of quadrature operators). Hence, in terms of $\cdot\Gamma^{(j)}_{k_1,l_1,\ldots,k_j,l_j}\cdot(\hat{\boldsymbol{a}},\hat{\boldsymbol{a}}^\dagger)$, supplementary equation (14) reads

$$\Gamma^{(j)}_{k_1,l_1,\ldots,k_j,l_j} = \left\langle \cdot\Gamma^{(j)}_{k_1,l_1,\ldots,k_j,l_j}\cdot(\hat{\boldsymbol{a}},\hat{\boldsymbol{a}}^\dagger)\right\rangle_{\varrho_\mathrm{P}}. \tag{105}$$

Note that for any fixed n, the anti-normal ordered expression (104) can be efficiently computed for all $j\in[n]$. Also note that normal and anti-normal ordered expressions of all powers of the number operator are explicitly given in ref.[15].

Then, by virtue of the *Optical Equivalence Theorem*[11–13], the expectation value of $\cdot\Gamma^{(j)}_{k_1,l_1,\ldots,k_j,l_j}\cdot(\hat{\boldsymbol{a}},\hat{\boldsymbol{a}}^\dagger)$ with respect to any preparation ϱ_P equals the expected value $\mathbb{E}_{Q_{\varrho_\mathrm{P}}}\left(\cdot\Gamma^{(j)}_{k_1,l_1,\ldots,k_j,l_j}\cdot\right)$ of $\cdot\Gamma^{(j)}_{k_1,l_1,\ldots,k_j,l_j}\cdot$ with respect to Q_{ϱ_P}. Here, Q_{ϱ_P} is understood as a classical phase-space probability distribution and $\cdot\Gamma^{(j)}_{k_1,l_1,\ldots,k_j,l_j}\cdot$ as a classical random variable that takes real values $\cdot\Gamma^{(j)}_{k_1,l_1,\ldots,k_j,l_j}\cdot(\boldsymbol{\alpha},\boldsymbol{\alpha}^*)$. $\cdot\Gamma^{(j)}_{k_1,l_1,\ldots,k_j,l_j}\cdot(\boldsymbol{\alpha},\boldsymbol{\alpha}^*)$ is defined as $\cdot\Gamma^{(j)}_{k_1,l_1,\ldots,k_j,l_j}\cdot(\hat{\boldsymbol{a}},\hat{\boldsymbol{a}}^\dagger)$ but replacing the operator vector $\hat{\boldsymbol{a}}$ by the complex vector $\boldsymbol{\alpha}\in\mathbb{C}^m$. That is, it holds that

$$\left\langle \cdot\Gamma^{(j)}_{k_1,l_1,\ldots,k_j,l_j}\cdot(\hat{\boldsymbol{a}},\hat{\boldsymbol{a}}^\dagger)\right\rangle_{\varrho_\mathrm{P}} = \int_{\boldsymbol{\alpha}\in\mathbb{C}^m} \cdot\Gamma^{(j)}_{k_1,l_1,\ldots,k_j,l_j}\cdot(\boldsymbol{\alpha},\boldsymbol{\alpha}^*)\,Q_{\varrho_\mathrm{P}}(\boldsymbol{\alpha},\boldsymbol{\alpha}^*)d^{2m}\boldsymbol{\alpha} =: \mathbb{E}_{Q_{\varrho_\mathrm{P}}}\left(\cdot\Gamma^{(j)}_{k_1,l_1,\ldots,k_j,l_j}\cdot\right), \tag{106}$$

where the short-hand notation $d^{2m}\boldsymbol{\alpha} := d^2\alpha_1\ldots d^2\alpha_m$ has been used.

Thus, in accordance with Supplementary Lemma 3, our experimental estimate $\Gamma^{(j)\star}_{k_1,l_1,\ldots,k_j,l_j}$ of $\Gamma^{(j)}_{k_1,l_1,\ldots,k_j,l_j}$ is now given by the finite-sample average

$$\Gamma^{(j)\star}_{k_1,l_1,\ldots,k_j,l_j} := \frac{1}{C_{\leq 2(n+1)}}\sum_{\kappa=1}^{C_{\leq 2(n+1)}} \cdot\Gamma^{(j)}_{k_1,l_1,\ldots,k_j,l_j}\cdot(\boldsymbol{\alpha}_\kappa,\boldsymbol{\alpha}^*_\kappa), \tag{107}$$

with $\boldsymbol{\alpha}_\kappa$ sampled from Q_{ϱ_P}. The integer $C_{\leq 2(n+1)}$ is the number of copies of the preparation devoted to the moment $\Gamma^{(j)}_{k_1,l_1,\ldots,k_j,l_j}$, as indicated in Supplementary Box 2, and is given explicitly in supplementary equation (85). The rest of the argument follows as in the Gaussian case. Note, finally, that the definition of the maximal variances $\sigma_{\leq 2(n+1)}$ remains the same too.

Supplementary Note 5 - Stability against systematic errors.

Apart from statistical errors, Arthur's measurement procedure could also have systematic errors. That is, if the characterisation of his single-mode measurement channels is erroneous, he could actually be measuring different observables from the ones he thinks he does. Theorems 2 and 3, as well as the Supplementary Corollary 1, consider only statistical errors, i.e., those that can be decreased by increasing the number of measurement repetitions (and, hence, the number of copies of ϱ_P). Since systematic errors cannot be decreased by accumulating statistics, no certification method based exclusively on the measurement statistics can rule them out. However, the stability analyses of Supplementary Lemmas 5, 8, and 11 hold regardless of the nature of errors. Thus, the experimental estimates $F^{(0)\star}$, $F^{(n)\star}$, and $F^{(n)\star}_{\mathcal{S}}$ (and, therefore, also the certification tests) turn out to be robust also against small systematic errors: The total fidelity deviations due to systematic errors scales linearly with the magnitude of the largest systematic error and polynomially in all the other relevant parameters as given in supplementary equations (52), (77), and (91).

Still, it is illustrative to consider a physically relevant example. A typical systematic error is non-unit quantum efficiency of the detectors used for homodyning. In that case, the probability density function $\tilde{\mathcal{P}}$ of measurement outcomes r of a quadrature $\hat{r}$ equals the ideal one $\mathcal{P}$ convolutioned with the normal distribution $\mathcal{N}$ of mean zero and squared variance $(1-\eta)/4\eta$, where η is the quantum efficiency of the detectors[9]. That is, $\tilde{\mathcal{P}}(r) = (\mathcal{P}*\mathcal{N})(r) := \int dr'\mathcal{P}(r')\mathcal{N}(r-r')$. Using that the first and second *non-central moments* of $\mathcal{N}$ satisfy

$$\langle r\rangle_{\mathcal{N}} := \int dr r\mathcal{N}(r-r') = r' \tag{108a}$$

and

$$\langle r^2\rangle_{\mathcal{N}} := \int dr r^2\mathcal{N}(r-r') = r'^2 + \frac{1-\eta}{4\eta}, \tag{108b}$$

respectively, one obtains that

$$\langle r\rangle_{\tilde{\mathcal{P}}} = \langle r\rangle_{\mathcal{P}} \tag{109a}$$

and

$$\langle r^2\rangle_{\tilde{\mathcal{P}}} = \langle r^2\rangle_{\mathcal{P}} + \frac{1-\eta}{4\eta}. \tag{109b}$$

That is, the expectation value of $\hat{r}$ is not affected by this type of systematic errors and that of $\hat{r}^2$ deviates from the ideal one by $(1-\eta)/(4\eta)$. Furthermore, the expectation values of products of quadrature operators acting on different modes are also not affected, as this type of systematic error acts independently on different modes.

In the absence of statistical errors, this leads to an error vector $\boldsymbol{\epsilon} = \mathbf{0}$ and an error matrix $\mathcal{E}^{(1)}$ that is diagonal and such that $\|\mathcal{E}^{(1)}\|_{\max} \leq (1-\eta)/(4\eta)$, so that $\|\mathcal{E}^{(1)}\|_1 \leq m(1-\eta)/(2\eta)$. Inserting this into supplementary equation (57), we see for instance that, for Gaussian targets, the contribution to the deviation of the fidelity estimate due to non-ideal detector efficiency in the homodyne detectors is smaller than $s_{\max}^2 m\frac{1-\eta}{2\eta}$. This, in turn, is smaller or equal than a desired constant maximal error ε if

$$\eta \geq \frac{s_{\max}^2 m}{2\varepsilon + s_{\max}^2 m} \approx 1 - \frac{2\varepsilon}{s_{\max}^2 m}, \tag{110}$$

where the approximation holds whenever $s_{\max}^2 m \gg 2\varepsilon$. The scaling given by the bound in supplementary equation (110) is experimentally convenient in that, in particular, it implies that the detector inefficiency $1-\eta$ needs to decrease only inversely proportional with the number of modes m.

Another typical systematic error is the limited power of the local oscillator field used for the homodyne detection: The homodyne (photocurrent difference) statistics, i.e., the distribution of homodyne measurement outcomes, match exactly the statistics of the corresponding quadrature only in the limit of an intense local-oscillator beam[10]. The most obvious difference is that the homodyne statistics is discrete whereas the quadrature statistics is continuous, with the former approximating the latter increasingly better as the local-oscillator power increases. However, we emphasise that our method relies on the estimation of only the expectation values of quadratures and not their full statistics. It can be seen that, provided that the local oscillator is in a coherent state, the effect of limited power is just to increase the variance of the effective quadrature without changing its expectation value with respect to the ideal case. Furthermore, in the multi-mode scenario, if the different modes are homodyned with independent local oscillators, the latter is also true for products of quadratures, as the ones considered in this work. Therefore, the effect of systematic errors due to limited homodyne local-oscillator power in our fidelity estimates is expected not to be critical either.

Supplementary Note 6 - Auxiliary mathematical relations.

Derivation of the properties of the operator valued Pochhammer-Symbol. We begin with equation (21a). The general relationship

$$(a_j^\dagger)^t \hat{n}_j (a_j)^t = p_t(\hat{n}_j), \tag{111}$$

for $t \in \mathbb{N}$, can be shown by induction starting from $p_0(\hat{n}_j) = \hat{n}_j$ and noting that, for all $t \geq -1$,

$$a_j^\dagger p_t(\hat{n}) a_j = a_j^\dagger \hat{n}_j(\hat{n}_j - 1)(\hat{n}_j - 2)\cdots(\hat{n}_j - t)a_j \tag{112}$$

$$= a_j^\dagger \hat{n}_j(\hat{n}_j - 1)(\hat{n}_j - 2)\cdots(\hat{n}_j - (t-1))a_j(\hat{n}_j - (t+1)) \tag{113}$$

$$= p_t(\hat{n}_j)(\hat{n}_j - (t+1)) \tag{114}$$

$$= p_{t+1}(\hat{n}_j), \tag{115}$$

as can be verified using the commutation relations between a_j and $a_j^\dagger$. Setting $t = n_j$ gives equation (21a).

In turn, equation (21b) can be shown by noting that

$$(a_j^\dagger)^{n_j}(a_j)^{n_j} = (a_j^\dagger)^{n_j-1}\hat{n}_j(a_j)^{n_j-1} \tag{116}$$

and applying supplementary equation (111), for $t = n_j - 1$, to the right-hand side of supplementary equation (116).

Proof of supplementary equation (46). Note that for $x = 0$ both sides of supplementary equation (46) yield 1 and hence the

bound holds in that case. We make the substitution $y = 1/x$ and show that the bound in supplementary equation (46) holds for all $x > 0$ by proving the following:

$$\frac{1}{1-\mathrm{e}^{-y}} \leq \frac{1}{y} + \frac{1}{2(1+1/y)} + \frac{1}{2} \quad \forall\, y \geq 0. \tag{117}$$

But this is equivalent to

$$2y^2 + 3y + 2 \leq \mathrm{e}^{y}(2+y). \tag{118}$$

A straightforward calculation shows that both sides and also the first derivatives of both sides coincide at $y = 0$, while the second derivative of the right hand side is always larger than the second derivative of the left hand side. This proves supplementary equation (117) and hence finishes the proof of the bound in supplementary equation (46).

Supplementary Refences.

[1] F. Dell'Anno, D. Buono, G. Nocerino, A. Porzio, S. Solimeno, S. De Siena, and F. Illuminati, *Tunable non-Gaussian resources for continuous-variable quantum technologies*, Phys. Rev. A **88**, 043818-043830 (2013).

[2] F. Dell'Anno, S. De Siena, L. Albano, and F. Illuminati, *Continuous-variable quantum teleportation with non-Gaussian resources*, Phys. Rev. A **76**, 022301-022311 (2007).

[3] C. Navarrete-Benlloch, R. García-Patrón, J. H. Shapiro, and N. J. Cerf, *Enhancing quantum entanglement by photon addition and subtraction*, Phys. Rev. A **86**, 012328-012336 (2012).

[4] P. Kok, W. J. Munro, K. Nemoto, T. C. Ralph, J. P. Dowling, and G. J. Milburn *Linear optical quantum computing with photonic qubits*, Rev. Mod. Phys. **79**, 135-174 (2007).

[5] E. Knill, R. Laflamme, and G. J. Milburn, *A scheme for efficient quantum computation with linear optics*, Nature **409**, 46-52 (2001).

[6] C. Weedbrook, S. Pirandola, R. Garcia-Patron, N. J. Cerf, T. C. Ralph, J. H. Shapiro, and S. Lloyd, *Gaussian quantum information*, Rev. Mod. Phys. **84**, 621-669 (2012).

[7] S. L. Braunstein, *Squeezing as an irreducible resource*, Phys. Rev. A **71**, 055801-055804 (2005).

[8] M. Reck, A. Zeilinger, H. J. Bernstein, and P. Bertani, *Experimental realization of any discrete unitary operator*, Phys. Rev. Lett. **73**, 58-61 (1994).

[9] A. Ferraro, S. Olivares, and M. G. A. Paris. *Gaussian states in continuous variable quantum information*, ISBN 88-7088-483-X (Bibliopolis, Napoli, 2005). Preprint at http://arxiv.org/abs/quant-ph/0503237.

[10] S. L. Braunstein, *Homodyne statistics*, Phys. Rev. A **42**, 474-481 (1990).

[11] M. O. Scully and M. S. Zubairy, *Quantum Optics*, Cambridge University Press (1997).

[12] H. Carmichael, *Statistical Methods in Quantum Optics 2: Non-Classical Fields*, Springer (2008).

[13] H. M. Wiseman and G. J. Milburn, *Quantum Measurement and Control*, Cambridge University Press (2010).

[14] G. M. D'Ariano, *Homodyning as universal detection*, in "Quantum Communication,Computing, and Measurement", Edited by O. Hirota, A. S. Holevo, and C. M. Caves, Plenum Publishing (New York and London 1997). Preprint at http://arxiv.org/abs/quant-ph/9701011.

[15] J. M. Vargas-Martínez and H. Moya-Cessa, *Normal and anti-normal ordered expressions for annihilation and creation operators*, Rev. Mex. Fís. E **52**, 13-16 (2006); http://arxiv.org/abs/1304.0385.
